# Decoding dissociation of sequence-specific protein–DNA complexes with non-equilibrium simulations

**DOI:** 10.1093/nar/gkad1014

**Published:** 2023-11-11

**Authors:** Thor van Heesch, Peter G Bolhuis, Jocelyne Vreede

**Affiliations:** Van ’t Hoff Institute for Molecular Sciences, University of Amsterdam, Netherlands; Van ’t Hoff Institute for Molecular Sciences, University of Amsterdam, Netherlands; Van ’t Hoff Institute for Molecular Sciences, University of Amsterdam, Netherlands

## Abstract

Sequence-specific protein–DNA interactions are crucial in processes such as DNA organization, gene regulation and DNA replication. Obtaining detailed insights into the recognition mechanisms of protein–DNA complexes through experiments is hampered by a lack of resolution in both space and time. Here, we present a molecular simulation approach to quantify the sequence specificity of protein–DNA complexes, that yields results fast, and is generally applicable to any protein–DNA complex. The approach is based on molecular dynamics simulations in combination with a sophisticated steering potential and results in an estimate of the free energy difference of dissociation. We provide predictions of the nucleotide specific binding affinity of the minor groove binding Histone-like Nucleoid Structuring (H-NS) protein, that are in agreement with experimental data. Furthermore, our approach offers mechanistic insight into the process of dissociation. Applying our approach to the major groove binding ETS domain in complex with three different nucleotide sequences identified the high affinity consensus sequence, quantitatively in agreement with experiments. Our protocol facilitates quantitative prediction of protein–DNA complex stability, while also providing high resolution insights into recognition mechanisms. As such, our simulation approach has the potential to yield detailed and quantitative insights into biological processes involving sequence-specific protein–DNA interactions.

## Graphical Abstract

**Graphical Abstract:**
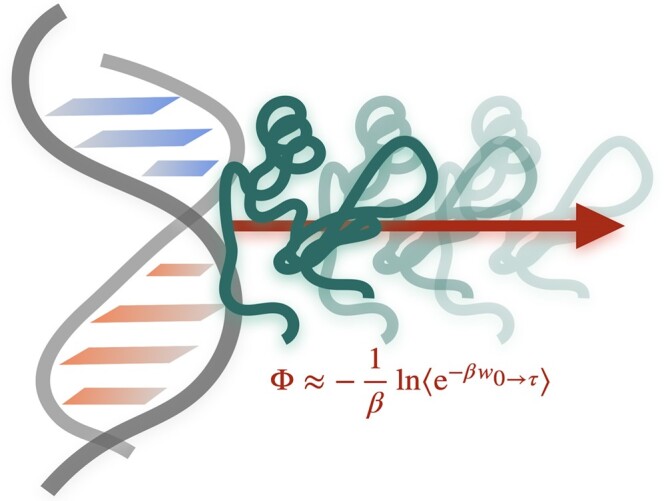

## Introduction

Sequence specificity in protein–DNA interactions plays a fundamental role in accessing genetic information (1). Processes such as gene regulation, DNA replication and DNA damage repair involve proteins binding to specific nucleotide sequences. The selectivity in complex formation is determined by the formation of specific intermolecular contacts. These interactions consist of electrostatics, hydrogen-bonds and steric considerations. Positively charged amino-acid side chains are strongly attracted to the negatively charged backbone of DNA. Hydrogen-bonds are formed between nucleobases, sugar groups and proteins. Examples of steric considerations are the widths of the major and minor grooves in dsDNA, but also deformations of ideal B-DNA and curvature. Understanding the mechanisms of selectivity in protein–DNA complexes would provide new insights into gene regulation, DNA organization and any other process involving interaction between proteins and nucleic acids (2).

Up to now, experiments cannot provide sufficient resolution in both space and time to obtain highly detailed atomistic insights into recognition mechanisms of protein–DNA complexation. Protein crystallography or NMR provide atomic resolution structures, albeit averaged over long time scales. Spectroscopic methods can offer high time resolution, however, with limited spatial information. All-atom molecular dynamics (MD) simulations can supplement these experimental methods by providing both atomic-level spatial and temporal resolution by tracking a molecular system in time. Still, quantitative predictions require the observation of many transitions from one (meta) stable state to another. The large system size of protein–DNA complexes, in combination with the slow interaction dynamics requires MD simulations in the order of milliseconds to seconds for statistically relevant quantitative predictions. These long timescales prohibit sampling of DNA binding and unbinding with current computational resources, allowing only for qualitative predictions. Even on the fastest supercomputers, which can simulate 100 μs per day (3), simulating a single transition event could take several days up to >27 years of wallclock time.

By adding an additional potential to drive the system along a reaction coordinate allows for enhanced sampling, i.e. exploring both more conformations and more transitions. This so-called biasing potential can be implemented in various ways in order to predict the free energy difference between the bound and unbound state of protein–DNA complexes, such as adaptive biasing force (4), metadynamics (5,6), alchemical sampling (7,8), umbrella sampling (9,10) and steered molecular dynamics (11). For such approaches to be successful the biasing potential has to resemble the reaction coordinate closely. If the biasing potential is sufficiently close to the underlying free energy surface, these approaches can provide a reliable estimate of the free energy, and can predict transition pathways (12). As a consequence, each protein–DNA complex requires a specific biasing potential, which may complicate the comparison of different systems. Moreover, studies that have not compared multiple DNA sequences cannot guarantee that the biasing method can adequately distinguish sequence-specific interactions and the associated recognition mechanisms. Here we present an efficient and generalisable simulation protocol to quantify the sequence specificity of protein–DNA complexes. We successfully apply the protocol to the Histone-like Nucleoid Structuring (H-NS) protein in complex with a high affinity AT-rich DNA sequence, and its GC-analogue, providing an explanation of the nucleotide specific binding affinity of H-NS.

H-NS is a bacterial DNA-binding protein, involved in DNA organization. Bacteria contain their genomic DNA in a distinct structure called the nucleoid. Organization of the nucleoid is mediated by several factors, including so-called architectural proteins. H-NS is such an architectural protein, and plays a key role in the genome organization of Gram-negative enterobacteria. Depending on external conditions H-NS structures DNA by forming filaments along DNA duplexes, either by binding to two separate DNA duplexes or to adjacent sites on the same duplex (13–18). Furthermore, H-NS prefers to bind to conserved nucleotide sequences that are AT-rich and tend to be curved (19–25). More specifically, experimental studies using either protein binding microarrays or chromatin immunoprecipitation uncovered that H-NS has a high affinity for AT-rich sequences with short A-tracts interrupted by TA steps (15,21,24,26). In addition, changing the relative location of high affinity H-NS binding sites on plasmids in relation to one another results in different plasmid and H-NS complex topologies (27). H-NS can adopt a roadblock function by binding near or at promoter regions and restructuring the accessibility of DNA, regulating transcription globally (28–30). In addition, H-NS functions as a xenogeneic silencer due to its preference to bind foreign genetic material, and is also associated with bacterial stress resistance and virulence through the activation of foreign H-NS repressed genes in response to lethal environmental conditions (26,31–33).

**Figure 1.**
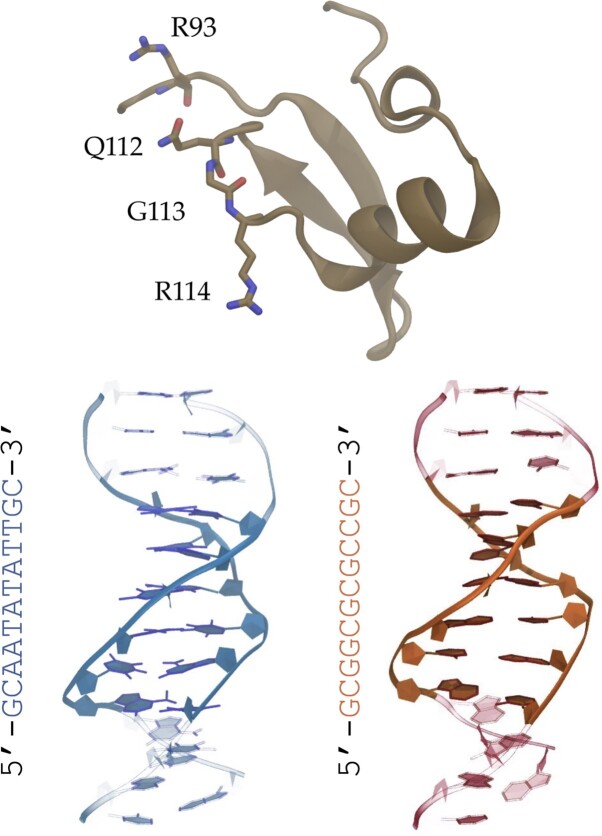
Snapshots of the components studied in this work. At the top is the DNA-binding domain of H-NS (brown) with the QGR motif (residues 112–114) and R93 shown as sticks. Below are the two dsDNA sequences with on the left the high affinity sequence (blue) and on the right its GC-analogue (orange).

The protein structure of H-NS comprises of 137 amino acid residues consisting of two domains: the oligomerization domain and the DNA binding domain (DBD). The first 83 residues constitute the oligomerization domain, containing two sites: a homodimerization site and a multimerization site to form higher order structures (34). At low concentrations, H-NS primarily exists as a dimer (13). The DBD is made up by residues 89-137, which consists of an anti-parallel β-sheet, an α helix and a 3_10_ helix (35,36). According to NMR experiments on the full H-NS protein, the oligomerization domain and DBD function independently of one another (37). This data further suggests that a flexible linker connects the two domains. The loop of DBD (residues 112-114) contains a conserved three amino acid sequence: QGR (16,37), see Figure 1 (top). This motif interacts with the minor groove of DNA in a comparable manner to other H-NS related proteins, such as Ler and Lsr2, according to NMR investigations (36,38,39).

Thus far, there is no quantitative information about how H-NS binds to the high affinity sites, and the recognition process is far from understood. Yet, such insights are essential to obtain an accurate estimate for the affinity of H-NS for different nucleotide sequences. In the first part of this paper, we use microsecond all-atom molecular dynamics simulations to characterize the difference in binding mode of the DNA-binding domain of H-NS upon changing from a high affinity sequence to a GC-analogue (i.e. change adenine to guanine and thymine to cytosine) see Figure 1 (bottom), and the effect of the minor groove shape on the stability of binding. In the second part of the paper, we provide mechanistic insights and a prediction of the dissociation potential of mean force of H-NS in complex with either a high affinity DNA or its GC-analogue sequence by means of steered MD simulations: a non-equilibrium molecular dynamics protocol that allows estimation of the free energy estimation by application of Jarzinsky’s equality (40,41). We find that H-NS binds stronger to AT-rich DNA, in agreement with experimental observations. Furthermore, we assess the limitations of this protocol with regard to the hyper-parameters used to determine the sequence-specificity of protein–DNA complexes. Finally, to confirm that our approach can indeed predict differences in binding strength for protein–DNA complexes, we applied our simulation protocol to the ETS domain of the PU.1 transcription factor in complex with three different nucleotide sequences. This protein contains a winged helix-turn-helix motif that binds to the major groove (42). We show that our protocol correctly predicts that the ETS domain–DNA complex with the strongest interaction indeed contains the sequence experimentally identified as the consensus sequence (43–45).

In conclusion, we present a fast molecular simulation approach to quantify interactions between proteins and DNA, facilitating comparison between different nucleotide sequences. We demonstrate our approach for the minor groove binding protein H-NS and the major groove binding ETS domain in complex with different nucleotide sequences, resulting in predictions of the dissociation free energy that are in agreement with experimental data. This approach can easily be extended to variations in the protein. Experimental validation could consist of protein–DNA binding assays based on fluorescence, i.e. by incorporating a fluorescent nucleobase (46) or in a Förster Resonance Energy Transfer (47) or nanofluidics set up (48). In a broader scope, simulations as presented in this work will yield detailed insights that can be compared to experiments directly, thus providing a valuable tool in the study of protein–DNA complexes.

## Materials and methods

### Molecular dynamics

We used two systems in this study, the DBD of H-NS (later referred to as H-NS) in complex with a high affinity dsDNA sequence and its GC-analogue. We performed Molecular Dynamics (MD) simulations of the following systems in explicit water: H-NS; dsDNA with nucleotide sequences 5’-GCAATATATTGC-3’ and 5’-GCGGCGCGCCGC-3’, and H-NS with the respective dsDNA nucleotide sequences. H-NS’s initial structure was taken from the solution NMR structure of the DNA-binding region of *Salmonella typhimurium* of H-NS-like protein Bv3F (residues 91–139, PDB code 2L93) (36). An acetyl cap was placed on the N-terminus to neutralize its charge, since this domain is connected to a linker in the full length protein. For dsDNA structure we chose as a high affinity sequence AATATATT based on known H-NS binding sites (15,21,24,26), containing two AT steps. This sequence is capped with GC base pairs at both ends to lower the probability of base opening at the DNA ends. Coordinates of H-NS bound to the minor groove of the high affinity 12-bp strand of dsDNA were obtained from earlier work (49), where several MD simulations were performed of this system, with the protein and DNA at at least 1 nm separation. These simulations enabled the charaterization of various binding modes, including one where the QGR motif was fully inserted into the minor groove. To obtain an initial conformation of H-NS bound to the GC-analogue sequence, the high affinity sequence with H-NS bound has been rebuild with Web 3DNA 2.0 by changing all adenines to guanines and thymines to cytosines while preserving the dsDNA backbone geometry (50).

Preparation of the system for molecular dynamics simulations consisted of placing the structures in a periodic dodecahedron box, with the box boundaries at least 1.0 nm from the system, followed by the addition of water molecules. The box size is at least 7 nm. To mimic experimental conditions (36) and neutralize the system, we added 50 mM NaCl by replacing water molecules with ions. Interactions between atoms are described by the AMBER14sb-parmbsc1 force field (51,52) in combination with the TIP3P water model (53). We selected this particular force field as it covers topologies for both amino acids and nucleotides, and provides good representations of the static and dynamic properties of DNA under a diverse range of conditions (52). For non-bonded interactions, both van der Waals and electrostatic, we used a cut-off at 1.1 nm. Long range electrostatic interactions were handled by the Particle Mesh Ewald method (54,55) with a grid spacing of 0.12 nm. To remove unfavorable interactions we performed energy minimization using steepest descent. By applying position restraints on the heavy atoms of the protein and DNA with a force constant in each direction of 1000 kJ/mol nm^2^ and performing 0.1 ns of MD at a temperature of 298 K and a pressure of 1 bar, we relaxed the water and ions around the initial structures.

After preparation, we performed multiple MD runs for the H-NS-DNA systems, varying initial conditions by assigning new random starting velocities drawn from the Maxwell-Boltzmann distribution at 298 K. See Table 1 for a summary of simulation times and systems. All simulations were performed with GROMACS, version 2020.4 (56,57) at a locally maintained cluster, with the leap-frog integration scheme and a time step of 2 fs, using LINCS (58) to constrain bonds in the protein and SETTLE (59) to constrain water bonds. All simulations were performed in the isothermal-isobaric ensemble at a pressure of 1 bar, using the v-rescale thermostat (60) and the isotropic Parrinello-Rahman barostat (61,62).

**Table 1.** Summary of MD simulations

System	Time	Runs	Total time
	(ns)	(#)	(μs)
DNA high affinity	1000	3	3.0
DNA GC-analogue	1000	3	3.0
H-NS & DNA high affinity in FI state	50	10	–
”	250	4	–
”	1000	2	3.5
H-NS & DNA GC-analogue in FI state	50	10	–
”	250	4	–
”	1000	2	3.5

Summary of the MD simulations in this work. Total time is the cumulative simulation time for each system.

### Steered molecular dynamics

We performed steered MD (SMD) calculations on both the H-NS bound to the high affinity and the GC-analogue sequence. The SMD simulations in this work were carried out using the open-source PLUMED library (63), version 2.6.3 (64) in combination with GROMACS, version 2020.4 (56,57). Preparation of the systems consisted of the exact same procedure as the MD simulations, except the box boundaries were set slightly larger, 1.2 nm instead of 1 nm distance between protein–DNA complex and the box boundary to counteract spurious periodic image problems during the pulling simulation. In addition, the pulling coordinate was computed without taking into account periodic boundary conditions. The pulling coordinate is based on distances between atom pairs in the protein and DNA. As the protein and DNA are pulled apart during the steered MD, the distance of an atom pair may be shorter when considering one atom in a neighboring periodic box, and therefore be considered instead of the distance between the atoms in the same box.

The minor groove of dsDNA contains mainly hydrogen bond acceptors (except for guanine), and the DNA binding motif in H-NS contains mainly hydrogen bonds donors. Previous work has identified a promising quantitative descriptor to follow the interaction between DNA and H-NS by counting the number of contacts between hydrogen bond acceptors in the minor groove of DNA, labeled *i* and hydrogen bond donors in the QGR motif of H-NS, labeled *j* (49). For each pair *ij* we define a contact *c*_*ij*_ with the expression:


(1) \begin{eqnarray*} c_{ij} = \left\lbrace \begin{array}{ll} 1 \qquad \qquad &{\rm if } (r_{ij}-d_0) < 0 \\ \frac{1-(\frac{r_{ij-d_0}}{r_0})^{nn}}{1-(\frac{r_{ij-d_0}}{r_0})^{mm}} \qquad \qquad &{\rm if } (r_{ij}-d_0)\ge 0 \end{array}\right., \end{eqnarray*}

where *r*_*ij*_ is the distance between atom *i* and atom *j*, located in the DNA and H-NS, respectively. The parameters *r*_0_ = 0.4 nm, *d*_0_ = 0.25 nm, *nn* = 2, *mm* = 4 have been chosen such to count contacts at hydrogen bond distance (<0.35 nm) as 1 and contacts at 0.7 nm as 0.5. This provides a smooth and descriptive function that can discriminate between the different binding modes. Summing all contacts for all pairs results in the contact map parameter *C*_*QGR*−*minor*_:


(2) \begin{eqnarray*} C_{QGR-minor}=\sum _{j=1}^{N_{H-NS}}\sum _{i=1}^{N_{DNA}}c_{ij}, \end{eqnarray*}

where *N*_*DNA*_ and *N*_*H*−*NS*_ are the number of interaction sites in the DNA and in H-NS. In this contact map, hydrogen bond donors in Q112, G113 and R114 have been included. In addition, we calculated the number of contacts between hydrogen bond donors in R93 (atoms N, NZ, NH1 and NH2) and hydrogen bond acceptors in the minor groove of the AT bases *C*_*R*93−*minor*_. To discriminate between different binding modes, a contact map parameter *c*_*j*_ is also computed for each hydrogen bond donor *j* in the QGR motif, separately, with respect to the hydrogen bond acceptors in the minor groove of the DNA:


(3) \begin{eqnarray*} C_{j}=\sum _{i=1}^{N_{DNA}}c_{ij}, \end{eqnarray*}

with *j* indicating the atoms Q112-N, Q112-NE2, G113-N, R114-N, R114-NZ, R114-NH1, R114-NH2 in the QGR motif. Note that any type of contact can be included. We decided to limit the number of contact points per nucleobase to one, to enable comparison between different sequences.

To steer the system along this contact map from the fully inserted (FI) state to a dissociated state the parameters in the switching function were adjusted to *r*_0_ = 3.0 nm, *d*_0_ = 0.3 nm, *nn* = 1, *mm* = 12. Now the contact map follows a linear trend instead of a rational decay, see Supplementary Figure S1. This modification ensures a constant displacement of each contact throughout the pulling simulation. The high contact count represents the FI state of H-NS bound to the DNA and the lower bound to a state of where H-NS is dissociated from the backbone of the DNA (BB). To distinguish between the two types of contact maps, we refer to the initial parameters resulting in smooth decay of the contact map as *C*_*QGR*−*minor*_, and the adjusted linear contact map used during the pulling of the steered MD simulations as λ. The final pulling coordinate, λ, is defined along the normalized linear contact map range with 1.0 referring to the FI state and 0.0 to the DNA dissociated BB state of the protein DNA pair. In the linear contact map space, λ, we steer the system from 108 to 65 contacts, which is equal to a contact count of 39 and 10 with the parameters used in a previous study (49) respectively.

For each system we performed 20 SMD simulations of 100 ns (unless noted otherwise), and obtained the corresponding work-λ curves. From these simulations, we can obtain an estimate of the free energy difference Δ*G* by computing the potential of mean force (PMF, indicated by the symbol Φ) from the exponential average work required for dissociation in each system by applying the Jarzynski equality (40) as:


(4) \begin{eqnarray*} \Phi \approx -\frac{1}{\beta }\ln \langle {\rm e}^{-\beta w_{0\rightarrow \tau }}\rangle , \end{eqnarray*}

where β = 1/*k*_*B*_*T*, with T the temperature and *k*_*B*_ Boltzmann’s constant, and the total work, *w*, is the sum of the work done in each interval along the initial state, 0, to the final state, τ.

As an error measure we also computed the standard deviation over all work-λ curves, which can be expressed as the square root of the variance and, when weighted with β as:


(5) \begin{eqnarray*} \sigma = \frac{\beta }{2}\sqrt{\langle w^{2} \rangle - \langle w \rangle ^{2}}. \end{eqnarray*}

Finally we define:


(6) \begin{eqnarray*} \Delta W \equiv W_{max} - W_{min}, \end{eqnarray*}

over the PMF as an estimate of the free energy difference between the H-NS–DNA complex *W*_*min*_ and free H-NS *W*_*max*_.

### Analysis

During the MD simulations, the frames were stored every 20 ps. The calculation of various geometric parameters based on the *C*_*QGR*−*minor*_ contact map described above and PMFs were computed with in-house Python scripts (65). In addition, we calculated the root mean square deviation (RMSD) of the DNA, with respect to equilibrated starting structures, including all atoms in the calculation with MDtraj python libary (66). We computed the minor groove width of the DNA according to the Curves+ definition (67). To visualize the dissociation mechanism, two-dimensional PMFs were generated by fitting a two-dimensional polynomial with the NumPy’s linalg.lstsq method (68). The fitted surface is constructed by projecting two coordinates, here the *C*_*Q*112−*minor*_ and *C*_*R*114−*minor*_, on the *x* and *y* axis. Next, we defined a grid on this surface by binning each coordinate. Here we used 100 bins for each axis. For each binned grid-point we collected all work values of the individual SMD runs and computed the Boltzmann weighted average work for each bin, giving the respective *z*-value of the surface. If no work values are found in the grid-point the bin is assigned a *z*-value above the maximum work observed in the SMD runs. Further analysis consisted of visual inspection and generation of snapshots in VMD (69).

## Results and discussion

### Stability of the H-NS DNA complexes

H-NS prefers to bind to AT-rich DNA (22–24,27) with a highly conserved motif in the DNA-binding domain consisting of three amino acids: QGR (16,37). In previous simulation work (49) we were able to identify three main H-NS binding modes based on interactions of the QGR motif with the minor groove of the DNA: bound to the DNA backbone (BB), with one side chain of the QGR motif inside the minor groove, referred to as partially inserted (PI) and with the entire QGR motif inside the minor groove, referred to as fully inserted (FI). In addition, the process of H-NS binding to AT-rich DNA resulting in the FI state was found to be rate-limiting compared to the non-specific association of H-NS to the DNA backbone. In this study we will be starting from the FI state to investigate the sequence specificity of the H-NS DNA Binding Domain (DBD). We compared a high affinity 12-bp dsDNA based on known H-NS binding sites (15,21,26,27) with a GC-analogue both capped with four GC repeats, resulting in the respective nucleotide sequences 5’-GCAATATATTGC-3’ and 5’-GCGGCGCGCCGC-3’. GC caps are added to prevent base opening at the DNA ends. The starting conformation of the H-NS DBD bound to the high affinity 12-bp strand was taken from earlier work (49). To obtain a FI starting structure for the GC-analogue, the H-NS bound high affinity sequence has been rebuilt with Web 3DNA 2.0 by changing all adenines to guanines and thymines to cytosines while preserving the dsDNA backbone geometry(50).

**Figure 2.**
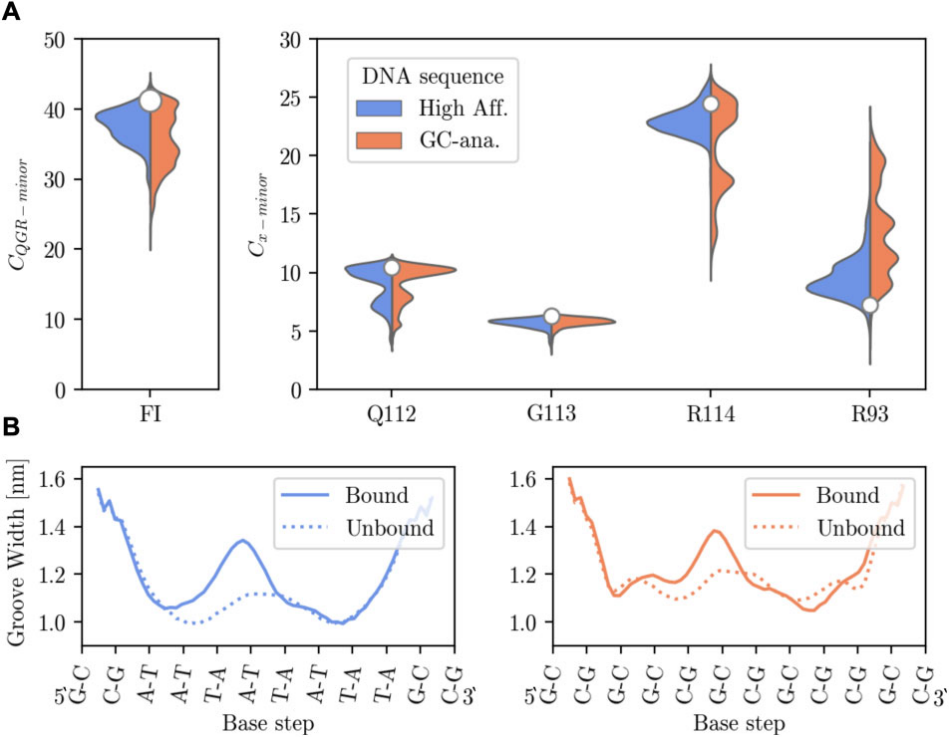
(**A**) Analysis of fully inserted (FI) state based on the cumulative MD simulations of 10× 50 ns, 4× 250 ns and 2× 1 μs. Violin plot of the *C*_*QGR*−*minor*_ distribution starting from the FI states of the high affinity (left wing and in blue) and GC-analogue (right wing and in orange). *C*_*QGR*−*minor*_ decomposed into the contribution of the individual residues, Q112, G113 and R114 respectively with the addition of minor groove contacts of R93. (**B**) The bottom left and right panel show the average minor groove width with H-NS bound (solid line) and without H-NS bound (dotted line) to the high affinity and GC-analogue sequence respectively.

Subsequently, we performed MD simulations of varying length to address the stability of the FI state for both systems. Figure 2 shows the comparative analysis of the two H-NS bound DNA sequences consisting of a contact analysis of the interacting H-NS residues and minor groove width measurements of the DNA with and without H-NS present. The first panel of Figure 2A shows a violin plot containing the probability histogram of *C*_*QGR*−*minor*_ of the FI states from the cumulative MD simulations with the FI state as the initial configuration. Here, the high affinity sequence in shown in blue on the left hand side and the GC-analogue in orange on the right hand side of the violin plot, and the white dot indicates the contact number of the starting structure of the high affinity sequence. Visual inspection of all trajectories as well as no *C*_*QGR*−*minor*_ values lower than 20, indicate no spontaneous dissociation of H-NS from neither DNA sequence has occurred. Furthermore, the shape of the *C*_*QGR*−*minor*_ distributions of the two sequences is different: a broader distribution with peaks between 30 and 40 is observed for the GC-analogue, while the high affinity sequence contains less variance with a high density peak at 39. The increased variance in the GC-analogue suggest the FI state is less stable than in the high affinity sequence.

By decomposing the *C*_*QGR*−*minor*_ count we investigated the contribution of individual residues in the binding motif, see Q112, G113 and R114 in Figure 2A. R114 populates multiple states ranging from 12 to 26 contact counts for the GC-analogue. Compared to the high affinity sequence in which R114 populates a state with a high contact count centered around 23, the arginine in the GC-analogue has more freedom to move and may be less tightly bound to the minor groove. In both sequences Q112 shows two modes, a sharp peak at 11 and a smaller and broader mode between 8 and 5 *C*_*Q*112−*minor*_ contacts, indicating that the residue has some freedom to move. Upon visual inspection the lower Q112 contacts correspond to interactions with the DNA backbone and high contact with the formation of H-bonds with the nucleobases. No significant difference is observed in the G113 contact count distributions. In both sequences R93 mainly forms contacts with the backbone and transient intramolecular contacts with Q112. The R93 contacts are higher in the GC-analogue, however these contacts are transient, lower (≤ 20 contacts), and do not occur in the high affinity system. Overall, the observations from the MD simulations indicate a less stable binding mode of the QGR motif upon mutation from a high affinity sequence to a GC-analogue, which is reflected by the lowered median, and broadening of the *C*_*QGR*−*minor*_ and *C*_*R114*−*minor*_ distributions.

In addition, we investigated if the DNA deforms or alters shape upon binding of H-NS by measuring the averaged minor groove width, the major groove width and base pair and base step parameters according to the curves+ definition (67). The major groove widths and the base pair and step parameters are shown in Supplementary Figures S2 and S3 respectively. These parameters show little change when comparing the bare DNA to the DNA in complex with H-NS. The bottom left and right panel of Figure 2B show respectively the minor groove width of the high affinity and GC-analogue nucleotide sequence with H-NS in the FI state in solid lines and without the presence of H-NS indicated by the dotted lines. The minor groove width of the unbound AT-rich sequence is completely symmetric and smooth. In contrast, changing the high affinity sequence to GC-analogue results in a more rugged profile and increases the minor groove width. In the FI state the QGR motif is bound to the most central base pairs with the glutamine oriented to the left (5′ end) and the arginine more to the right (3′ end) of the horizontal plot. The binding of the QGR motif causes widening of the minor groove where the Q112 is situated. This effect is observed for both sequences, although to a lesser extent in the GC-analogue. Arginine residues often bind to narrow minor grooves, since a narrow minor groove strongly enhances the negative electrostatic potential of the DNA (70). Our simulation shows that the R114 residue prefers to bind at the point where the width of the minor groove is smallest. This is due to the fact that A-tracts and AT-rich sequences tend to narrow the minor groove, while GC base pairs have a tendency to widen it. A wider minor groove could create more room for the protein to move. In the simulation of the GC-rich DNA we do indeed observe more fluctuations of the R114 contact count compared to AT-rich DNA.

### Dissociation of the complex

Since the *C*_*QGR*−*minor*_ contact maps in the FI state sample a comparable range of contact counts and for neither sequence H-NS has dissociated from the DNA, we selected 20 random configurations where the *C*_*Q112*−*minor*_ and *C*_*R114*−*minor*_ contact count was 10.25 ± 0.125 and 23 ± 0.25 respectively as input for the steered molecular dynamics (SMD) simulations. We measured the cumulative work required to dissociate H-NS along the linear contact map, λ, as the pulling coordinate described in the methods section. Using Jarzynski’s equality (40,41,71) we could turn the non-equilibrium work-dissociation curves into equilibrium free energy. In addition, we computed the standard deviation of all the individual work-dissociation curves as an error measure.

**Figure 3.**
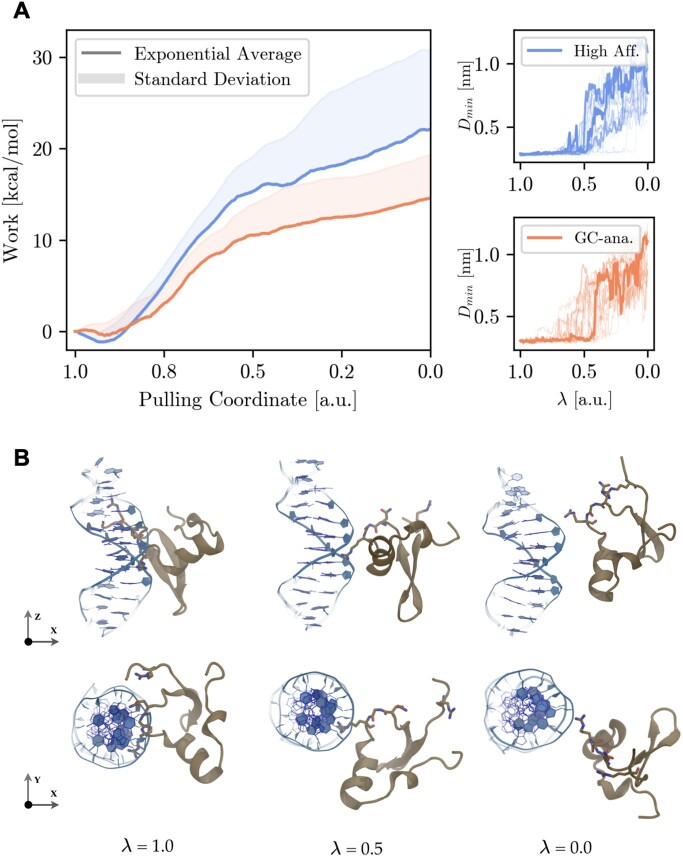
(**A**) PMF along *C*_*QGR*−*minor*_ showing the Boltzmann-weighted average work plot (solid lines) for each system (blue high affinity and orange GC-analogue) with the standard deviation of all work-curves as the shaded area above the Boltzmann-weighted average work. The two right panels show the minimum distance between the minor groove hydrogen bond acceptors and the hydrogen bond donors of the QGR binding motif for the respective systems. (**B**) Shows snapshots along the progression of the pulling coordinate at λ = 1.0, 0.5 and 0.0 for the lowest work SMD run of the high affinity sequence starting in the FI state (with the dsDNA in blue and H-NS in brown).

A comparison between the potential of mean force (PMF) of the dissociation of H-NS from the high affinity sequence and GC-analogue is shown in Figure 3A, which provides more insight into the nature of sequence specific protein–DNA dissociation. Compared to the GC-analogue the high affinity sequence has a steeper slope during the transition from the fully inserted state (λ = 1.0) to a partially inserted state (λ = 0.5). To reach a completely dissociated state in which H-NS makes only non-specific contacts with the backbone (λ = 0.0), the PMF shows a similar slope for both nucleotide sequences. Dissociation of H-NS from the high affinity sequence requires almost twice as much work (23.27 kcal/mol) compared to the GC-analogue (15.05 kcal/mol), resulting in a sequence specific difference ΔΔW of 8.22 kcal/mol. The two right panels of Figure 3A show the minimum distance of the QGR motif’s donor atoms with respect to the minor groove acceptor atoms (D_min_) for each individual SMD simulation run along the pulling coordinate. Up to 0.5 of the pulling coordinate the minimum distance remains constant at 0.3 nm (roughly coinciding with an hydrogen-bond distance) indicating the QGR motif has not left the minor groove crevice. From this point onward the slope at which the work curve increases also starts to decline, marking that the first major free energy barrier has been crossed when all residues in the binding motif no longer form H-bonds and solely interact with the DNA backbone. At the end of the pulling simulation the D_min_ has reached a value of at least 1.0 nm, which is enough space for water molecules to come in between the DNA and protein. Panel B of Figure 3 shows for the high affinity sequence snapshots of the lowest work SMD trajectory along the pulling coordinate at the FI state (λ = 1.0) to a state disconnected from the DNA ). At λ = 0.5 the R114 is still partially inserted into the minor groove, but at λ = 0.0 no direct contacts are visible between H-NS and the minor groove of the DNA. Our SMD simulations are thus able to differentiate the high affinity and GC-analogue binding of the H-NS, supporting the AT-rich DNA preference of H-NS.

**Figure 4.**
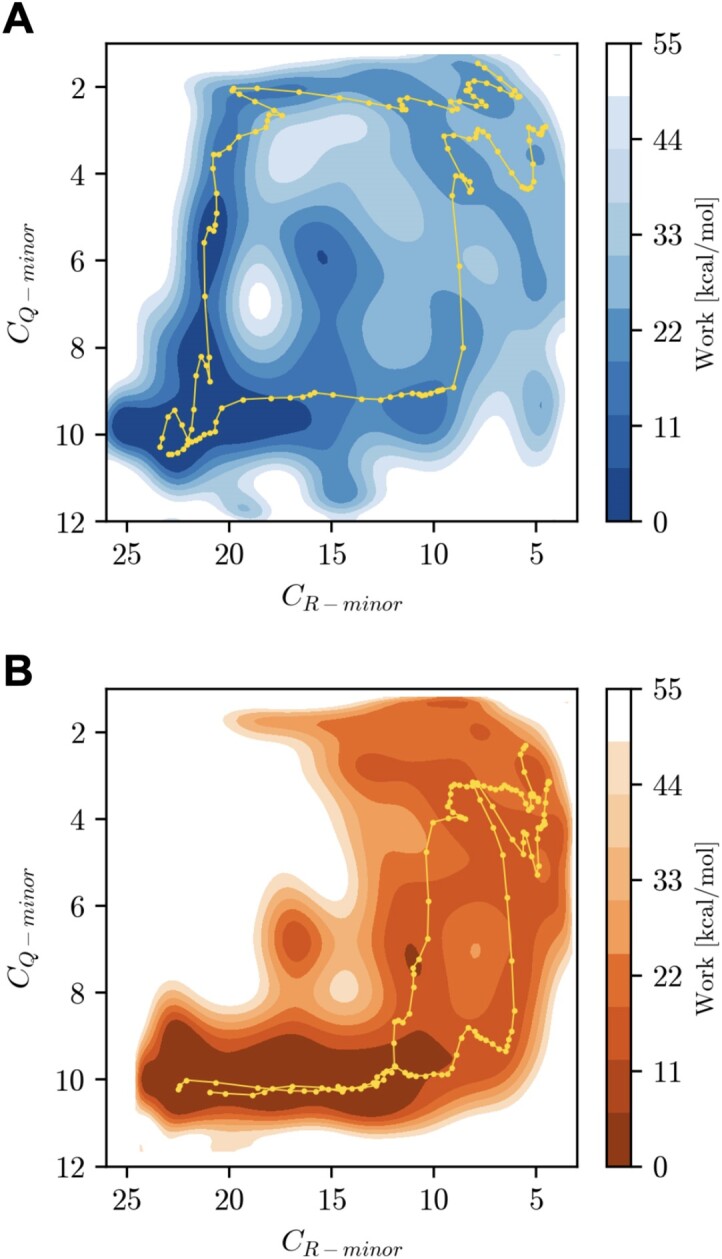
Panels **A** and **B** show the boltzmann-weighted 2D PMF along the Q112 and R114 contact count for the systems, panel A show the projection for the high affinity sequence (blue) and panel B for the GC-analogue (orange). In addition, the paths of the two lowest work runs are projected on the surface of the respective systems with the runs shown in yellow in panel A and green in panel B.

The exponential average of work and the standard deviation in Figure 3A coincide almost perfectly for both sequences in the range of 1.00 to 0.55 of the pulling coordinate, confirming the reliability of the PMF. The variance in the work curve starts to increase in the last regime of the pulling coordinate (≥0.55 a.u.). For the high affinity sequence, the variance is higher. Visual inspection of the pulling trajectories indicate variation in the order of dissociation of the QGR motif (see Figure 2) and may explain the origin of the higher variance. To explore this variation the Boltzmann averaged work is projected on the Q112 and R114 contacts, see Figure 4, panels A an B for the high affinity and GC-analogue sequence respectively. In addition, we show the two lowest work paths in each 2D-PMF. This projection allows us to investigate the dissociation mechanism of the binding motif. In panel A of Figure 4, we observe that dissociation can follow two directions, either Q112 starts to separate from the minor groove followed by R114 (denoted Q-G-R), or vice versa (denoted R-G-Q).

For the GC-analogue the dissociation mechanism starts exclusively with the detachment of R112 from the groove, after which Q112 completes the dissociation of the DBD from DNA, as seen in panel B of Figure 4. The observation that the dissociation pathway of the GC-analogue only follows the R-Q-G route, could be related to the higher variance observed of the *C*_*R*−*minor*_ in the FI MD simulations (Figure 2B). The increased flexibility of the R114 and the R-G-Q dissociation route can explain the comparatively flat slope of the initial part of the PMF of the GC-analogue as seen in Figure 3A. The hypothesis that the widening of the minor groove width weakens the electrostatic interactions of the R114 with the nucleobases, and enhances with the narrowing of the minor groove plays an effect in the binding strength, has now been further supported by the PMFs obtained via the SMD simulations. In addition, we show that it is much more likely that for both sequences the mechanism follows a sequential dissociation of the QGR binding motif .

Since we have established two dissociation pathways for the high affinity sequence, we can further investigate the origin of the high variance by categorizing the individual work curves based on whether Q112 or R114 starts dissociating first. We consider a path as R-G-Q when the *C*_*Q*−*minor*_ ≤ 5 and Q-G-R if *C*_*Q*−*minor*_ ≥ 5 when measured at *C*_*QGR*−*minor*_ equals 30 contacts, the individual work curves are shown in Supplementary Figure S4A. The variance in the R-G-Q route is much lower and frequent compared to the Q-G-R path (14 versus 6 runs). When comparing the PMFs of both routes, the initial phase is consistent, however, when the slope of the PMF curve decreases starting from 0.4 a.u. the variation in the work-curves begins to increase significantly in the Q-G-R route. The work curves seem to correlate with fluctuations in the shape of the DNA strand. Indeed, when we compute the root-mean-squared deviation of the DNA with respect to equilibrated ideal B-DNA (*RMSD*_*DNA*_) for each individual SMD run based on the initial frame of the run, the high work curves also have an increased *RMSD*_*DNA*_ (≥ 0.4 nm), especially when the dissociation pathway follows the Q-G-R route. Visual inspection of high work runs shows that starting from λ = 0.4, the R114 residue does not dissociate. Instead, R114 pulls the central portion of the DNA strand along, causing the ends of the DNA to bend away from H-NS. Upon final detachment of R114, hydrogen bonds in multiple base pairs break, resulting in extremely high work and RMSD values for the DNA. This process is illustrated in Supplementary Figure S4B. Note that the high work runs have a negligible contribution to the Boltzmann-weighted PMF and are not representative for the dissociation mechanism. To summarize, SMD runs with high work result from deformation of the DNA, caused by residues sticking to DNA during the dissociation process.

**Figure 5.**
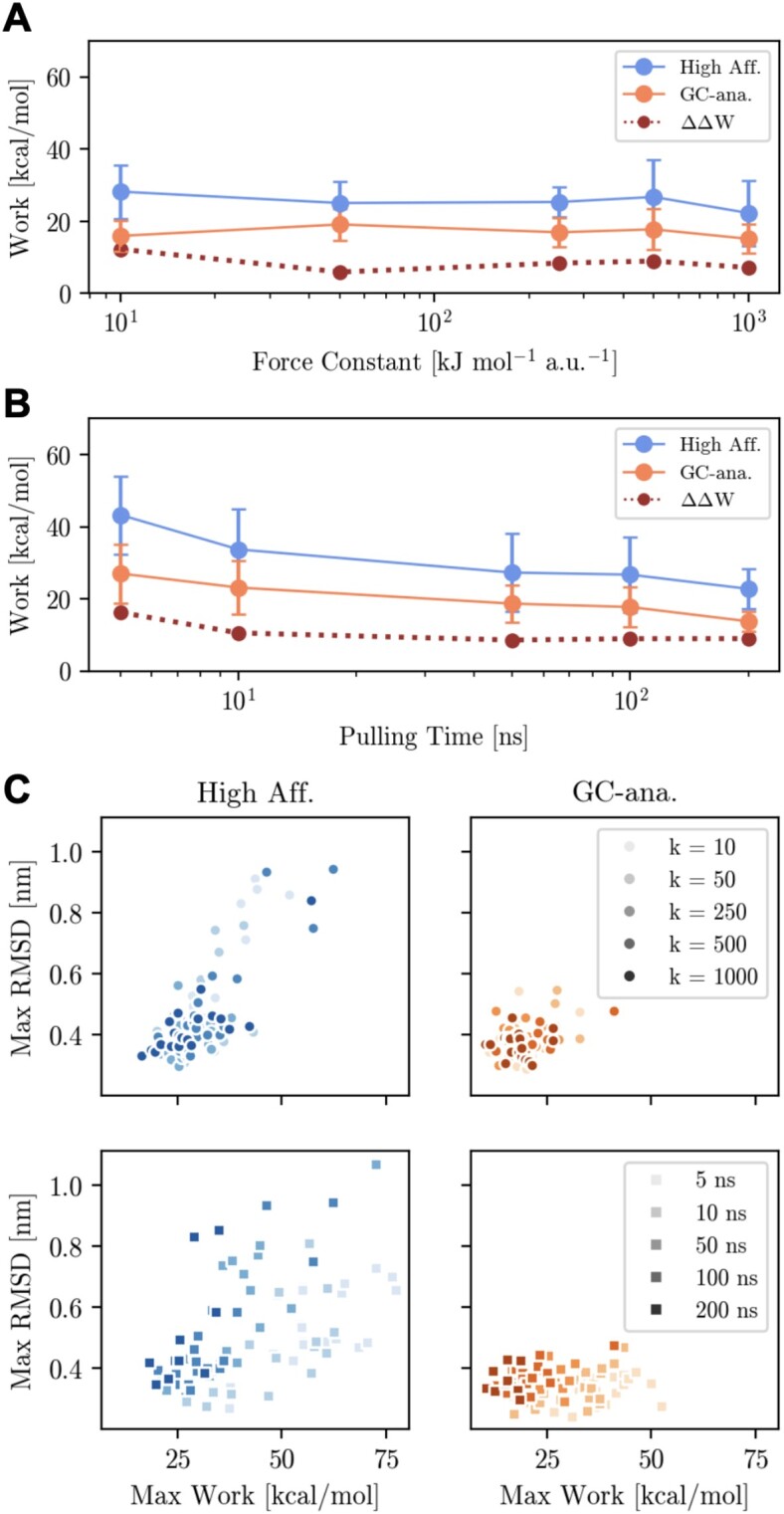
(**A**) Benchmark of force constant at 100 ns. (**B**) Benchmark of pulling speed at 500 kJ/mol. (**C**) Scatterplots of maximum RMSD_*DNA*_ with respect of the maximum work value of each individual SMD run. Note that all estimates were obtained based on 20 SMD runs, except with a pulling rate of 200 ns where we carried out only 10 runs for each system. The Pearson product-moment correlation coefficient in the high affinity force constant scatterplot is 0.77, and 0.51 in the pulling speed scatterplot. For the GC-analogue force constant scatterplot correlation coefficient between the maximum work and the maximum *RMSD*_*DNA*_ is 0.32, and 0.05 in the pulling speed scatterplot.

To further assess the quality of the results obtained above, we carried out the same protocol for both sequences with different force constants and pulling velocities during the pulling simulations. Figure 5A shows the Δ*W* values of the PMFs for both systems (in blue the high affinity sequence and orange the GC-analogue) as well as the ΔΔ*W* (dark brown and dotted lines). The ΔΔ*W* is the difference between PMF of dissociation of the two sequences defined as ΔΔ*W* ≡ |Δ*W*_*A*_−Δ*W*_*B*_|, with *A* and *B* being the respective systems. The top panel shows the values for different force constants of 10, 50, 250, 500 and 1000 kJ/mol respectively, and a pulling time of 100 ns. When doubling the force constant from 500 to 1000 kJ/mol, the ΔW values decrease only slightly for both systems, but show the same sequence specific difference and the change of the ΔΔ*W* is still within the same margin of error. By lowering the force constant of 500 kJ/mol by a factor of 0.5 also does not change the trend and ΔΔ*W* value. However, lowering the force constant one and two orders of magnitude further, the prediction starts to deviate from the previous values. With a force constant of 50 kJ/mol the difference between the systems becomes smaller and with a force constant of 10 kJ/mol the difference between the two systems becomes larger. A force constant that is too low will result in overestimation of the work. In the range of 250 to 1000 kJ/mol the estimation of the work remains the same. The bottom panel shows the values for different pulling times of 5, 10, 50, 100 and 200 ns respectively, with the force constant fixed at 500 kJ/mol. Generally, pulling faster results in an overestimation of the PMF values, however, already starting from 10 ns the ΔΔ*W* values seem to have converged. Pulling slower does seem to reduce the error in the PMFs of the systems. Other aspects could also affect the quality of the prediction, such as the choice of force field. However, this would result in a systematic error that and not an increase in statistical errors.

Previously we indicated a correlation between high work SMD runs and increased RMSD values of DNA. To further examine this relationship, we plotted the maximum work and maximum observed RMSD values of DNA for each SMD run in panel B of Figure 5 The results show that this correlation holds for the high affinity sequence, regardless of the force constant and pulling time, with correlation coefficients of 0.77 and 0.51, respectively. However, the maximum work values are nearly half as low in the GC-analogue and the RMSD values are almost a factor of 2.5 lower. These low values result in correlation coefficients that are close to zero (0.05 for the pulling speed benchmark) or indicate only a weak correlation (0.32 for the force constant benchmark) in the GC-analogue plots. Increased GC content is known to result in more stable DNA due to more favourable stacking interactions (72). This could explain the lower level of DNA deformation observed in the SMD simulations of the GC-analogue compared to the high affinity sequence. Overall, the benchmark shows that our protocol to address the sequence-specificity of protein DNA complexes displays robustness with respect to the hyper-parameters used during the SMD simulations.

Choosing an appropriate pulling coordinate for the steered MD is essential for proper sampling of the transition from bound H-NS to unbound H-NS closer to the underlying free energy surface. Here we require the pulling coordinate to be sufficiently general for multiple nucleotide sequences and therefore applicable to any protein–DNA complex. Coordinates based on centers of mass do not have enough resolution, hence we opted for the contacts between hydrogen bond acceptors in the minor (or major) groove of DNA and hydrogen bond donors in the protein. Such a contact map is thus already a quantitative indicator of binding strength, while centers of mass only provide relative distance between the groups. Supplementary Figure S5 compares for the high affinity sequence and GC-analogue the *C*_*QGR*−*minor*_ with different center of mass distance based metrics; H-NS and DNA (*COM*_*DBD*−*DNA*_), 4 central bases pairs and the QGR motif (*COM*_*cDNA*−*QGR*_), and the hydrogen bond acceptors in the minor groove and hydrogen bond donors in the QGR motif (*COM*_*A*−*D*_). Especially for the GC-analogue, a single COM mode aligns with multiple *C*_*QGR*−*minor*_ modes. Fixed centers of mass are insensitive to displacement along base pairs and different QGR conformations, and thus, cannot properly reflect the bound states properly. On the contrary, the contact map does provide a smooth and continuous descriptive function capable of discriminating different binding modes. In this example, we decided to begin the pulling process for both systems at the same contact count, thus using conformations with a high contact count of 39. For the GC-analogue, multiple conformations can be found at different *C*_*QGR*−*minor*_ values, i.e., not only in a high contact count state (as shown in Figure 2A). Starting from high contact values for both systems will result in the greatest amount of work and serves as a lower bound for the difference in free energy between sequences. If conformations with lower contact values are more prevalent, the difference in free energy between sequences will increase. For instance, if the pulling simulations for the GC-analogue were to start at 25 contacts, the difference in free energy between the two sequences would become even greater. Furthermore, the contact map can be easily extended to include transient contacts or additional contacts. The pulling coordinate in our protocol allows for comparison between systems, while retaining sufficient resolution and allows for the exploration of different mechanisms. Experimental validation could consist of protein–DNA binding assays based on fluorescence, i.e. by incorporating a fluorescent nucleobase (46) or in a Förster Resonance Energy Transfer (47) or nanofluidics set up (48).

To substantiate the claim that our simulation protocol can be applied to characterize the interaction between a protein and DNA, and quantify the affinity of a protein for different nucleotide sequences, we applied our approach to a major groove binding protein. The ETS domain of the PU.1 transcription factor binds the major groove of DNA with a winged helix-loop-helix motif (73) and recognizes purine-rich sequences containing a 5’-(A/T)GGA(A/T)-3’ consensus, see Supplementary Figure S6. Equilibrium titration experiments (43) as well as alchemical calculations (7) quantified the binding of the ETS domain to its consensus sequence as well as various other nucleotide sequences including an anti-consensus sequence. We performed 1 μs long MD simulations on the ETS domain in complex with three nucleotide sequences, and computed the free energy difference of the dissociation of the complex. A detailed description of this study is provided in the Supplementary Information, with Supplementary Table S1 listing the atoms included in the contact map, Supplementary Figure S7 showing the results from the MD simulations and Supplementary Figure S8 showing the results from the SMD simulations. Our results are in the same order of magnitude of the experimental values, as listed in Supplementary Table S2 in the SI. This means, we were able to confirm that the ETS domain binds stronger to the consensus sequence in comparison to a (A to G, and T to C) inverted sequence and the experimentally determined anti-consensus sequence. Performing this analysis in our current set up required about a week of simulation time on a mixed GPU (RTX 3090)/CPU (AMD) architecture. The main adjustment to the protocol outlined for H-NS in this work involve three aspects: adjust the contact map to the system under investigation, identify from which range in the contact map to select starting configurations for the SMD simulations, and over which range of the contact map the pulling simulations should run. The first aspect warrants some discussion, as a larger contact map slows down the calculation. Adding one extra atom to the contact map increases the number of pairwise computations increases with a factor equal to the number of atoms, thus slowing down the steered MD simulations. This is currently also a technical hardware limitation, if the bias force calculation is vectorized, this part of the calculation can be also done on the GPU and removes the extra overhead caused by the CPU-GPU communication latency. Note that with a too small the contact map, comparison of different sequences is no longer possible.

We expect that our simulation protocol is generally able to quantify the binding strengths and characterize the dissociation mechanism of protein–DNA complexes with the current settings. The MD simulations will provide the contacts required for the contact map based steered MD simulations, which can run with the settings for the length of the simulations and the force constant we report here. We expect that the simulation length and the force constant have to be adjusted when applying our protocol to systems that are much larger, such as the Lac repressor, or to systems that make many more contacts, such as proteins enveloping the DNA, such as enzymes involved in DNA replication or repair.

## Conclusion

We present a fast simulation protocol to determine the potential of mean force of the dissociation of protein–DNA complexes. Our approach is able to differentiate between different nucleotide sequences. We demonstrate the quality of our protocol by quantifying the sequence specific binding of H-NS to DNA. The difference of the potential mean force of dissociation between a high affinity sequence and its GC-analogue is predicted to be 8.22 kcal/mol. Our SMD simulations are thus able to differentiate sequence-specific protein binding. These results support experimental evidence that H-NS prefers to bind to AT-rich DNA. In addition, our protocol provides mechanistic insight into the dissociation process, finding that both sequences follow a sequential dissociation of the QGR binding motif. In particular the high affinity sequence showed strong binding of both Q112 and R114, resulting in multiple dissociation pathways. In contrast, the GC-analogue only showed one main dissociation route via R114 followed by Q112, confirming weaker binding of R114 to the GC-rich sequence. Furthermore, molecular dynamics simulations revealed sequence specific minor groove narrowing that resulted in more stable binding of the high affinity sequence. The simulation protocol displays robustness with respect to the hyper-parameters used in the SMD simulations. The presented approach facilitates quantitative prediction of the stability of protein–DNA complexes, thus opening up high resolution insights into DNA organization, gene regulation, DNA replication and other biological processes involving sequence specific protein–DNA interactions. We validated the claim that our protocol can successfully predict sequence dependent stability of protein–DNA complexes by applying our approach to the major groove binding ETS domain of the eukaryotic transcription factor PU.1. Simulations as presented in this work will yield detailed insights that can be compared to experiments directly, thus providing a valuable tool in the study of protein–DNA complexes.

Input files and simulation data are available on figshare: 10.6084/m9.figshare.c.6446950.

## Supplementary Material

gkad1014_Supplemental_File

## Data Availability

The data underlying this article are available in Figshare at https://doi.org/10.6084/m9.figshare.c.6446950.

## References

[1] von Hippel P.H. From ‘simple’ DNA-protein interactions to the macromolecular machines of gene expression. Annu. Rev. Biophys. Biomol. Struct. 2007; 36:79. 17477836 10.1146/annurev.biophys.34.040204.144521 PMC2660389

[2] Liu L.A. , Bradley P. Atomistic modeling of protein–DNA interaction specificity: progress and applications. Curr. Opin. Struct. Biol. 2012; 22:397–405. 22796087 10.1016/j.sbi.2012.06.002 PMC3425445

[3] Shaw[Liu D.E. , Adams[Liu P.J., Azaria[Liu A., Bank[Liu J.A., Batson[Liu B., Bell[Liu A., Bergdorf[Liu M., Bhatt[Liu J., Butts[Liu J.A., Correia[Liu T. et al. Anton 3: twenty microseconds of molecular dynamics simulation before lunch. Proceedings of the International Conference for High Performance Computing, Networking, Storage and Analysis. 2021; 1–11.

[4] Yonetani Y. , Kono H. Dissociation free-energy profiles of specific and nonspecific DNA–protein complexes. J. Phys. Chem. B. 2013; 117:7535–7545. 23713479 10.1021/jp402664w

[5] Furini S. , Domene C. DNA recognition process of the lactose repressor protein studied via metadynamics and umbrella sampling simulations. J. Phys. Chem. B. 2014; 118:13059–13065. 25341013 10.1021/jp505885j

[6] Singh R.K. , Mukherjee A. Molecular mechanism of the intercalation of the SOX-4 protein into DNA inducing bends and kinks. J. Phys. Chem. B. 2021; 125:3752–3762. 33848164 10.1021/acs.jpcb.0c11496

[7] Gapsys V. , de Groot B.L. Alchemical free energy calculations for nucleotide mutations in protein–DNA complexes. J. Chem. Theor. Comput. 2017; 13:6275–6289. 10.1021/acs.jctc.7b00849 29125747

[8] Gapsys V. , Khabiri M., de Groot B.L., Freddolino P.L. Comment on ‘deficiencies in molecular dynamics simulation-based prediction of protein–DNA binding free energy landscapes’. J. Phys. Chem. B. 2018; 124:1115–1123. 33236911 10.1021/acs.jpcb.0c10359

[9] Merino F. , Bouvier B., Cojocaru V. Cooperative DNA recognition modulated by an interplay between protein-protein interactions and DNA-mediated allostery. PLoS Comput. Biol. 2015; 11:e1004287. 26067358 10.1371/journal.pcbi.1004287 PMC4465831

[10] Wieczór M. , Czub J. How proteins bind to DNA: target discrimination and dynamic sequence search by the telomeric protein TRF1. Nucleic Acids Res. 2017; 45:7643–7654. 28633355 10.1093/nar/gkx534 PMC5737604

[11] Jakubec D. , Vondrasek J. Efficient estimation of absolute binding free energy for a homeodomain–dna complex from nonequilibrium pulling simulations. J. Chem. Theor. Comput. 2020; 16:2034–2041. 10.1021/acs.jctc.0c00006 32208691

[12] Bussi G. , Laio A. Using metadynamics to explore complex free-energy landscapes. Nat. Rev. Phys. 2020; 2:200–212.

[13] Falconl M. , Gualtierl M., La Teana A., Losso M., Pon C. Proteins from the prokaryotic nucleoid: primary and quaternary structure of the 15-kD Escherichia coli DNA binding protein H-NS. Mol. Microbiol. 1988; 2:323–329. 3135462 10.1111/j.1365-2958.1988.tb00035.x

[14] Williams R.M. , Rimsky S. Molecular aspects of the E. coli nucleoid protein, H-NS: a central controller of gene regulatory networks. FEMS Microbiol. Lett. 1997; 156:175–185. 9513262 10.1111/j.1574-6968.1997.tb12724.x

[15] Dame R.T. , Wyman C., Goosen N. H-NS mediated compaction of DNA visualised by atomic force microscopy. Nucleic Acids Res. 2000; 28:3504–3510. 10982869 10.1093/nar/28.18.3504 PMC110753

[16] Dorman C.J. H-NS: a universal regulator for a dynamic genome. Nat. Rev. Microbiol. 2004; 2:391–400. 15100692 10.1038/nrmicro883

[17] Liu Y. , Chen H., Kenney L.J., Yan J. A divalent switch drives H-NS/DNA-binding conformations between stiffening and bridging modes. Genes Dev. 2010; 24:339–344. 20159954 10.1101/gad.1883510 PMC2816733

[18] van der Valk R.A. , Vreede J., Qin L., Moolenaar G.F., Hofmann A., Goosen N., Dame R.T. Mechanism of environmentally driven conformational changes that modulate H-NS DNA-bridging activity. Elife. 2017; 6:e27369. 28949292 10.7554/eLife.27369 PMC5647153

[19] Yamada H. , Yoshida T., Tanaka K.-I., Sasakawa C., Mizuno T. Molecular analysis of the Escherichia coli has gene encoding a DNA-binding protein, which preferentially recognizes curved DNA sequences. Mol. Gen. Genet. 1991; 230:332–336. 1745240 10.1007/BF00290685

[20] Owen-Hughes T.A. , Pavitt G.D., Santos D.S., Sidebotham J.M., Hulton C.S., Hinton J.C., Higgins C.F. The chromatin-associated protein H-NS interacts with curved DNA to influence DNA topology and gene expression. Cell. 1992; 71:255–265. 1423593 10.1016/0092-8674(92)90354-f

[21] Lucchini S. , Rowley G., Goldberg M.D., Hurd D., Harrison M., Hinton J. C.D. H-NS mediates the silencing of laterally acquired genes in bacteria. PLoS Pathog. 2006; 2:e81. 16933988 10.1371/journal.ppat.0020081 PMC1550270

[22] Lang B. , Blot N., Bouffartigues E., Buckle M., Geertz M., Gualerzi C.O., Mavathur R., Muskhelishvili G., Pon C.L., Rimsky S. et al. High-affinity DNA binding sites for H-NS provide a molecular basis for selective silencing within proteobacterial genomes. Nucleic Acids Res. 2007; 35:6330–6337. 17881364 10.1093/nar/gkm712 PMC2094087

[23] Bouffartigues E. , Buckle M., Badaut C., Travers A., Rimsky S. H-NS cooperative binding to high-affinity sites in a regulatory element results in transcriptional silencing. Nat. Struct. Mol. Biol. 2007; 14:441–448. 17435766 10.1038/nsmb1233

[24] Ulissi U. , Fabbretti A., Sette M., Giuliodori A.M., Spurio R. Time-resolved assembly of a nucleoprotein complex between Shigella flexneri virF promoter and its transcriptional repressor H-NS. Nucleic Acids Res. 2014; 42:13039–13050. 25389261 10.1093/nar/gku1052 PMC4245942

[25] Navarre W.W. H-NS as a defence system. Bacterial chromatin. 2010; Springer 251–322.

[26] Navarre W.W. , Porwollik S., Wang Y., McClelland M., Rosen H., Libby S.J., Fang F.C. Selective silencing of foreign DNA with low GC content by the H-NS protein in Salmonella. Science. 2006; 313:236–238. 16763111 10.1126/science.1128794

[27] Japaridze A. , Renevey S., Sobetzko P., Stoliar L., Nasser W., Dietler G., Muskhelishvili G. Spatial organization of DNA sequences directs the assembly of bacterial chromatin by a nucleoid-associated protein. J. Biol. Chem. 2017; 292:7607–7618. 28316324 10.1074/jbc.M117.780239 PMC5418058

[28] Atlung T. , Ingmer H. H-NS: a modulator of environmentally regulated gene expression. Mol. Microbiol. 1997; 24:7–17. 9140961 10.1046/j.1365-2958.1997.3151679.x

[29] Rimsky S. Structure of the histone-like protein H-NS and its role in regulation and genome superstructure. Curr. Opin. Microbiol. 2004; 7:109–114. 15063845 10.1016/j.mib.2004.02.001

[30] Ono S. , Goldberg M.D., Olsson T., Esposito D., Hinton J.C., Ladbury J.E. H-NS is a part of a thermally controlled mechanism for bacterial gene regulation. Biochem. J. 2005; 391:203–213. 15966862 10.1042/BJ20050453 PMC1276917

[31] Oshima T. , Ishikawa S., Kurokawa K., Aiba H., Ogasawara N. Escherichia coli histone-like protein H-NS preferentially binds to horizontally acquired DNA in association with RNA polymerase. DNA Res. 2006; 13:141–153. 17046956 10.1093/dnares/dsl009

[32] Dorman C.J. H-NS, the genome sentinel. Nat. Rev. Microbiol. 2007; 5:157–161. 17191074 10.1038/nrmicro1598

[33] Forrester W.C. , Epner E., Driscoll M.C., Enver T., Brice M., Papayannopoulou T., Groudine M. A deletion of the human beta-globin locus activation region causes a major alteration in chromatin structure and replication across the entire beta-globin locus. Genes Dev. 1990; 4:1637–1649. 2249769 10.1101/gad.4.10.1637

[34] Arold S.T. , Leonard P.G., Parkinson G.N., Ladbury J.E. H-NS forms a superhelical protein scaffold for DNA condensation. Proc. Natl. Acad. Sci. U.S.A. 2010; 107:15728–15732. 20798056 10.1073/pnas.1006966107 PMC2936596

[35] Shindo H. , Iwaki T., Ieda R., Kurumizaka H., Ueguchi C., Mizuno T., Morikawa S., Nakamura H., Kuboniwa H. Solution structure of the DNA binding domain of a nucleoid-associated protein, H-NS, from Escherichia coli. FEBS Lett. 1995; 360:125–131. 7875316 10.1016/0014-5793(95)00079-o

[36] Gordon B.R. , Li Y., Cote A., Weirauch M.T., Ding P., Hughes T.R., Navarre W.W., Xia B., Liu J. Structural basis for recognition of AT-rich DNA by unrelated xenogeneic silencing proteins. Proc. Natl. Acad. Sci. U.S.A. 2011; 108:10690–10695. 21673140 10.1073/pnas.1102544108 PMC3127928

[37] Dorman C.J. , Hinton J.C., Free A. Domain organization and oligomerization among H-NS-like nucleoid-associated proteins in bacteria. Trends Microbiol. 1999; 7:124–128. 10203842 10.1016/s0966-842x(99)01455-9

[38] Cordeiro T.N. , Schmidt H., Madrid C., Juárez A., Bernadó P., Griesinger C., García J., Pons M. Indirect DNA readout by an H-NS related protein: structure of the DNA complex of the C-terminal domain of Ler. PLoS Pathog. 2011; 7:e1002380. 22114557 10.1371/journal.ppat.1002380 PMC3219716

[39] Ali S.S. , Xia B., Liu J., Navarre W.W. Silencing of foreign DNA in bacteria. Curr. Opin. Microbiol. 2012; 15:175–181. 22265250 10.1016/j.mib.2011.12.014

[40] Jarzynski C. Equilibrium free-energy differences from nonequilibrium measurements: a master-equation approach. Phys. Rev. E. 1997; 56:5018.

[41] Park S. , Schulten K. Calculating potentials of mean force from steered molecular dynamics simulations. J. Chem. phys. 2004; 120:5946–5961. 15267476 10.1063/1.1651473

[42] Sharrocks A. The ETS domain transcritpion factor family. Nat. Rev. 2001; 2:827–837. 10.1038/35099076 11715049

[43] Poon G. , MacGregor R. Jr Base coupling in sequence-specific site recognition by the TeS domain of murine PU.1. J. Mol. Biol. 2003; 328:805–819. 12729756 10.1016/s0022-2836(03)00362-0

[44] Li S.L. , Schlegel W., Valente A.J., Clark R.A. Critical flanking sequences of PU.1 binding sites in myeloid-specific promoters. J. Biol. Chem. 1999; 274:32453–32460. 10542290 10.1074/jbc.274.45.32453

[45] Gross P. , Yee A.A., Arrowsmith A.H., Macgregor R.B. J. Quantitative hydroxyl radical footprinting reveals cooperative interactions between DNA-binding subdomains of PU.1 and IRF4. Biochemistry. 1998; 38:9802–9811. 10.1021/bi9731448 9657694

[46] Jones A.C. , Neely R.K. 2-aminopurine as a fluorescent probe of DNA conformation and the DNA–enzyme interface. Quart. Rev. Biophys. 2015; 48:244–279. 10.1017/S0033583514000158 25881643

[47] Dey B. , Thukral S., Krishnan S., Chakrobarty M., Gupta S., Manghani C., Rani V. DNA–protein interactions: methods for detection and analysis. Mol. Cell. Biochem. 2012; 365:279–299. 22399265 10.1007/s11010-012-1269-z

[48] Frykholm K. , Müller V., Sriram K., Dorfman K.D., Westerlund F. DNA in nanochannels: theory and applications. Quart. Rev. Biophys. 2022; 55:e12. 10.1017/S0033583522000117 36203227

[49] Riccardi E. , Van Mastbergen E.C., Navarre W.W., Vreede J. Predicting the mechanism and rate of H-NS binding to AT-rich DNA. PLoS Comput. Biol. 2019; 15:e1006845. 30845209 10.1371/journal.pcbi.1006845 PMC6424460

[50] Li S. , Olson W.K., Lu X.-J. Web 3DNA 2.0 for the analysis, visualization, and modeling of 3D nucleic acid structures. Nucleic Acids Res. 2019; 47:W26–W34. 31114927 10.1093/nar/gkz394 PMC6602438

[51] Maier J.A. , Martinez C., Kasavajhala K., Wickstrom L., Hauser K.E., Simmerling C. ff14SB: improving the accuracy of protein side chain and backbone parameters from ff99SB. J. Chem. Theor. Comput. 2015; 11:3696–3713. 10.1021/acs.jctc.5b00255 PMC4821407 26574453

[52] Ivani I. , Dans P.D., Noy A., Pérez A., Faustino I., Hospital A., Walther J., Andrio P., Goñi R., Balaceanu A. et al. Parmbsc1: a refined force field for DNA simulations. Nat. Methods. 2016; 13:55–58. 26569599 10.1038/nmeth.3658 PMC4700514

[53] Jorgensen W.L. , Chandrasekhar J., Madura J.D., Impey R.W., Klein M.L. Comparison of simple potential functions for simulating liquid water. J. Chem. phys. 1983; 79:926–935.

[54] Cheatham T.I. , Miller J., Fox T., Darden T., Kollman P. Molecular dynamics simulations on solvated biomolecular systems: the particle mesh Ewald method leads to stable trajectories of DNA, RNA, and proteins. J. Am. Chem. Soc. 1995; 117:4193–4194.

[55] Essmann U. , Perera L., Berkowitz M.L., Darden T., Lee H., Pedersen L.G. A smooth particle mesh Ewald method. J. Chem. phys. 1995; 103:8577–8593.

[56] Van Der Spoel D. , Lindahl E., Hess B., Groenhof G., Mark A.E., Berendsen H.J. GROMACS: fast, flexible, and free. J. Comput. Chem. 2005; 26:1701–1718. 16211538 10.1002/jcc.20291

[57] Abraham M.J. , Murtola T., Schulz R., Páll S., Smith J.C., Hess B., Lindahl E. GROMACS: high performance molecular simulations through multi-level parallelism from laptops to supercomputers. SoftwareX. 2015; 1:19–25.

[58] Hess B. , Bekker H., Berendsen H.J., Fraaije J.G. LINCS: a linear constraint solver for molecular simulations. J. Comput. Chem. 1997; 18:1463–1472.

[59] Miyamoto S. , Kollman P.A. Settle: an analytical version of the SHAKE and RATTLE algorithm for rigid water models. J. Comput. Chem. 1992; 13:952–962.

[60] Bussi G. , Donadio D., Parrinello M. Canonical sampling through velocity rescaling. J. Chem. phys. 2007; 126:014101. 17212484 10.1063/1.2408420

[61] Parrinello M. , Rahman A. Polymorphic transitions in single crystals: a new molecular dynamics method. J. Appl. Phys. 1981; 52:7182–7190.

[62] Nosé S. , Klein M. Constant pressure molecular dynamics for molecular systems. Mol. Phys. 1983; 50:1055–1076.

[63] PLUMED consortium Promoting transparency and reproducibility in enhanced molecular simulations. Nat. Methods. 2019; 16:670–673. 31363226 10.1038/s41592-019-0506-8

[64] Tribello G.A. , Bonomi M., Branduardi D., Camilloni C., Bussi G. PLUMED 2: new feathers for an old bird. Comput. Phys. Commun. 2014; 185:604–613.

[65] Van Rossum G. , Drake F.L. Python 3 reference manual. 2009; Scotts Valley, CA CreateSpace.

[66] McGibbon R.T. , Beauchamp K.A., Harrigan M.P., Klein C., Swails J.M., Hernández C.X., Schwantes C.R., Wang L.-P., Lane T.J., Pande V.S. MDTraj: a modern open library for the analysis of molecular dynamics trajectories. Biophys. J. 2015; 109:1528–1532. 26488642 10.1016/j.bpj.2015.08.015 PMC4623899

[67] Lavery R. , Moakher M., Maddocks J.H., Petkeviciute D., Zakrzewska K. Conformational analysis of nucleic acids revisited: Curves+. Nucleic Acids Res. 2009; 37:5917–5929. 19625494 10.1093/nar/gkp608 PMC2761274

[68] Harris C.R. , Millman K.J., van der Walt S.J., Gommers R., Virtanen P., Cournapeau D., Wieser E., Taylor J., Berg S., Smith N.J. et al. Array programming with NumPy. Nature. 2020; 585:357–362. 32939066 10.1038/s41586-020-2649-2 PMC7759461

[69] Humphrey W. , Dalke A., Schulten K. VMD: visual molecular dynamics. J. Mol. Graph. 1996; 14:33–38. 8744570 10.1016/0263-7855(96)00018-5

[70] Rohs R. , West S.M., Sosinsky A., Liu P., Mann R.S., Honig B. The role of DNA shape in protein–DNA recognition. Nature. 2009; 461:1248–1253. 19865164 10.1038/nature08473 PMC2793086

[71] Park S. , Khalili-Araghi F., Tajkhorshid E., Schulten K. Free energy calculation from steered molecular dynamics simulations using Jarzynski’s equality. J. Chem. phys. 2003; 119:3559–3566.

[72] Yakovchuk P. , Protozanova E., Frank-Kamenetskii M.D. Base-stacking and base-pairing contributions into thermal stability of the DNA double helix. Nucleic Acids Res. 2006; 34:564–574. 16449200 10.1093/nar/gkj454 PMC1360284

[73] Kodandapani R. , Pio F., Ni C.-Z., Piccialli G., Klemsz M., McKercher S., Maki R.A., Ely K.R. A new pattern for helix–turn–helix recognition revealed by the PU. l ETS–domain–DNA complex. Nature. 1996; 380:456–460. 8602247 10.1038/380456a0

