# Supplementary material for: Decoding dissociation of sequence-specific protein–DNA complexes with non-equilibrium simulations
Source: Nucleic Acids Res. 2023 Nov 11;51(22):12150–60. doi: 10.1093/nar/gkad1014 (PMC10711434; doi:10.1093/nar/gkad1014)
Supplement: gkad1014_Supplemental_File [file gkad1014_supplemental_file.pdf]

Supplementary Information for: Decoding Dissociation of Sequence-Specific Protein-DNA Complexes with Non-equilibrium Simulations

Thor van Heesch [1], Peter G. Bolhuis [1] Jocelyne Vreede [1*]
[1]Van 't Hoff Institute for Molecular Sciences, University of Amsterdam, The Netherlands

September 26, 2023

Contents

1 Supplemental figures **2**
1.1 Contact map 2
1.2 Analysis of DNA conformation 3
1.3 Analysis of Steered Molecular Dynamics simulations 5

2 Example input file for Steered Molecular Dynamics **7**

3 Sequence specificity of the ETS domain **8**
3.1 Structure and function of ETS 8
3.2 Characterization of the ETS-DNA complexes 9
3.3 Nucleotide specificity of the ETS domain 11

*To whom correspondence should be addressed. Tel: +31 205256489; Email: j.vreede@uva.nl

1 Supplemental figures

1.1 Contact map

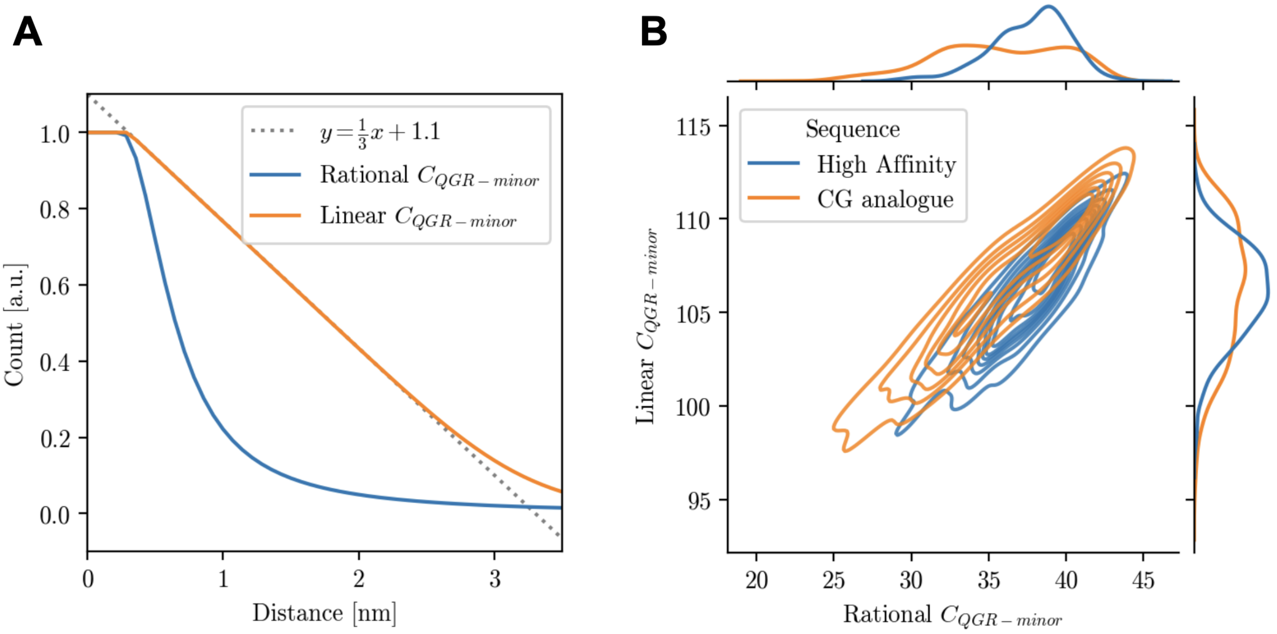

Supplementary Figure S1: A) Example of switching function used in the contact map with two different sets of parameters: the rational decay with $r_0 = 0.4$, $d_0 = 0.25$, $nn = 2$, $mm = 4$ in blue and the linear decay with $r_0 = 3.0$, $d_0 = 0.3$, $nn = 1$, $mm = 12$ in orange, and in gray a linear function for comparison. B) 2D kernel density estimates of the rational contact count, $C_{QGR-minor}$, with respect to the linear contact count for H-NS in complex with the high affinity sequence and its GC-analogue respectively based on the FI MD simulations (3.5 μs cumulative simulation time).

1.2 Analysis of DNA conformation

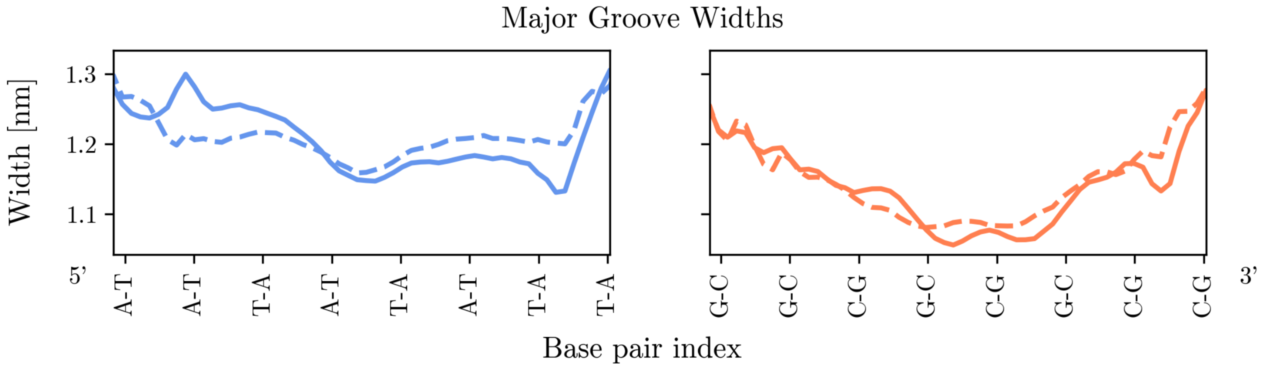

Supplementary Figure S2: Major groove widths of the high affinity sequence in blue and the GC-analogue in orange with the dotted line the bare DNA without H-NS bound, and the solid line with H-NS bound. Note, the GC-caps are not included in this plot of the major groove.

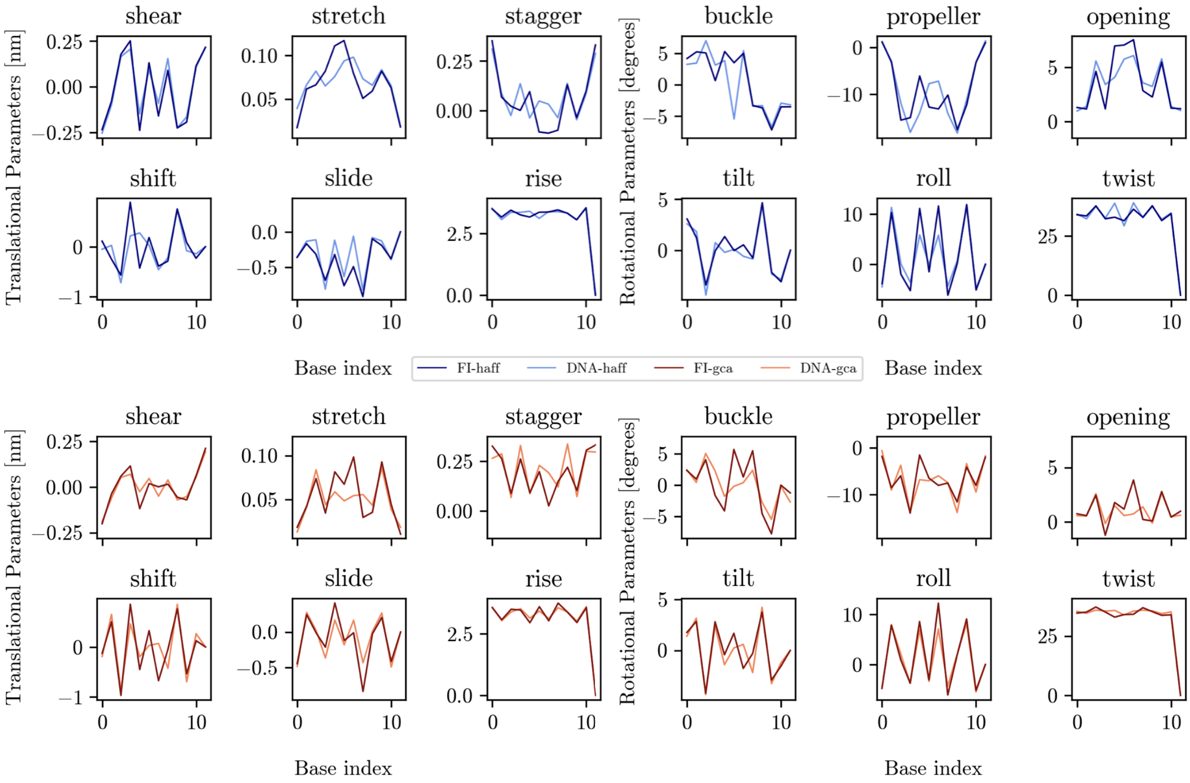

Supplementary Figure S3: Structural analysis of the DNA based on the MD simulations: Average base pair and base step parameters of the high affinity and GC-analogue systems with the dark blue and dark red colors representing the fully inserted (FI) system with H-NS bound and the light blue and orange color the parameters for the DNA without of H-NS. The base pair and base stp parameters are omputed according to the definition in Ref. as defined in Ref. [8].

1.3 Analysis of Steered Molecular Dynamics simulations

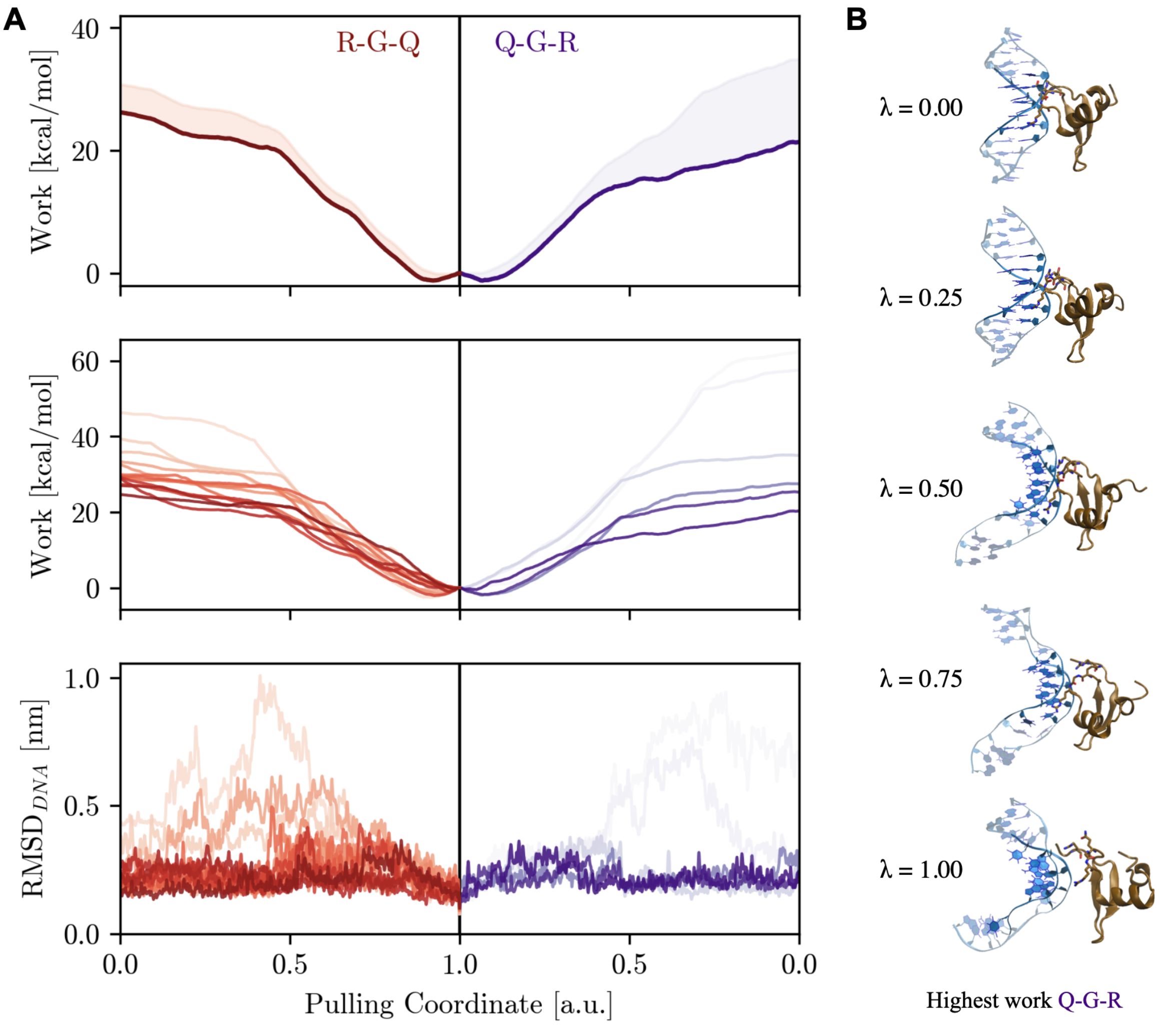

Supplementary Figure S4: A) Pulling coordinate with respect to the PMF, work of the individual SMD runs and $RMSD_{DNA}$ split based on the dissociation route of the high affinity system with left the R-G-Q path and right the Q-G-R path. The color gradient in the middle and lower panel correspond to the maximum work of the respective run (dark low work, light high work). B) Illustration of H-NS with the high affinity sequence along the pulling coordinate, λ, of the highest work SMD run following the Q-G-R route. Starting from $\lambda = 0.4$, the R114 residue does not dissociate. Instead, R114 pulls the central portion of the DNA strand along, causing the ends of the DNA to bend away from H-NS. Upon final detachment of R114, hydrogen bonds in multiple base pairs break, resulting in extremely high work and RMSD values for the DNA. Note that the runs with high work have a negligible contribution to the PMF.

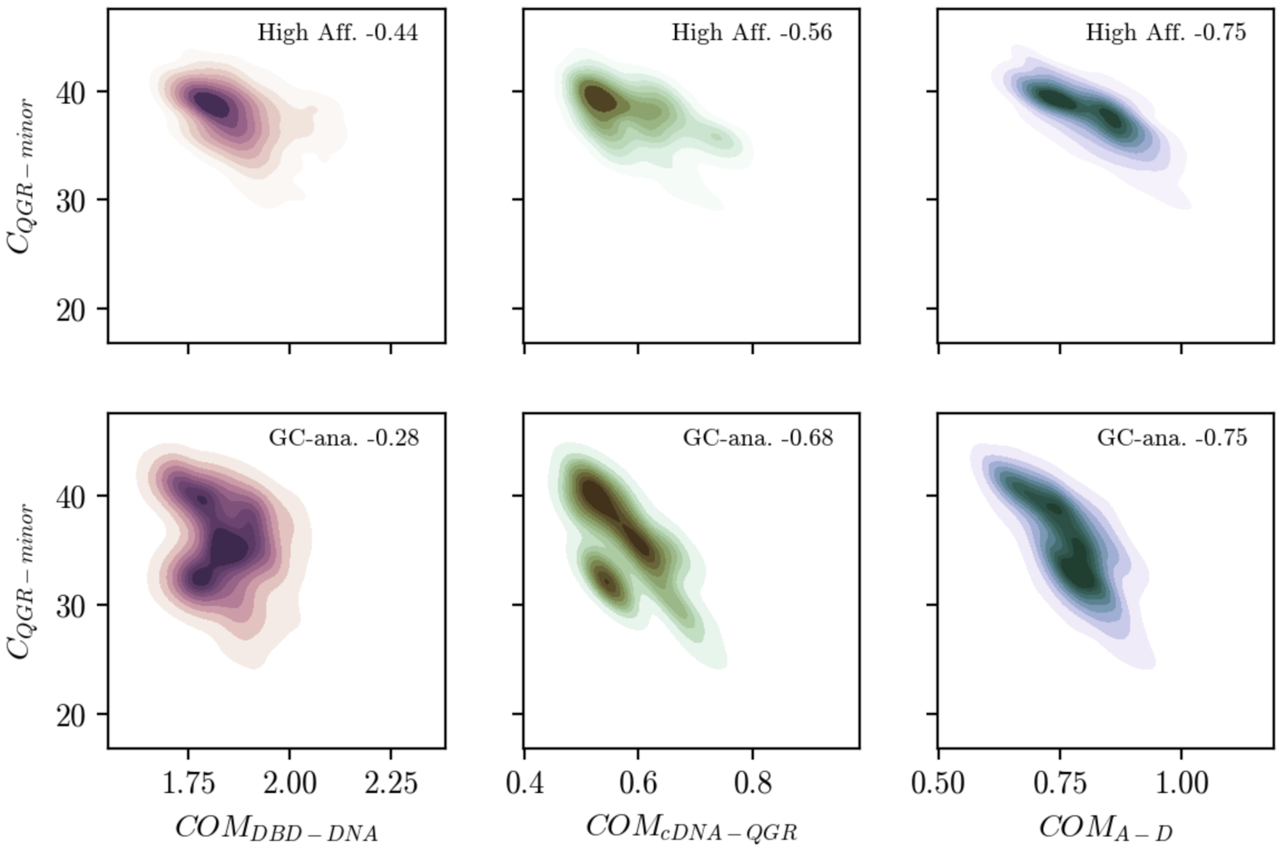

Supplementary Figure S5: 2D kernel density estimates (KDE) of the $C_{QGR-minor}$ contact count with respect to the center of mass distance in nm between: H-NS and DNA ($COM_{DBD-DNA}$), 4 central bases pairs and the QGR motif ($COM_{cDNA-QGR}$), and the hydrogen bond acceptors in the minor groove and hydrogen bond donors in the QGR motif (COM_{A-D}). The top row shows the KDEs of the high affinity sequence and the bottom row for the CG-analogue. In addition in each panel annotated with the correlation coefficient between the $C_{QGR-minor}$ and respective COM

2 Example input file for Steered Molecular Dynamics

Example input file to perform the steered molecular dynamics simulations using plumed[2, 12] and gromacs [13, 1]:

```
# Setting units
UNITS LENGTH=nm TIME=ps ENERGY=kj/mol
# System information
# Note that with this input file the file system.pdb must be provided
MOLINFO MOLTYPE=protein STRUCTURE=system.pdb
# Define the range of atoms for which periodic boundary effects must be removed
# Note that pbc effects are removed only for the calculation of the CV
# and that i and j represent the atom indices that include the protein-DNA complex
WHOLEMOLECULES ENTITY0=i-j

# Definition of the contactmap consising of pairwise distances
# The labels a_0, a_n, and b_0, b_n indicate atom numbers.
# The labels starting with a are atoms in the protein, ranging from 0 to n
# The labels starting with b are atoms in the DNA, ranging from 0 to m
cmap: CONTACTMAP ...
    ATOMS1=a_0,b_0
    ATOMSn=a_n,b_m
    SWITCH={RATIONAL R_0=3 D_0=0.3 NN=1 MM=12}

    SUM
    ...
# Steering plan reducing the total number of contacts from 108 to 65
# With KAPPA indicating the force constant of the spring in kJ/mol nm^2
MOVINGRESTRAINT ...
     ARG=cmap
     STEP0=0          AT0=108 KAPPA0=0
     STEP1=250000     AT1=108 KAPPA1=500
     STEP2=50250000   AT2=65 KAPPA2=500
    ...

# Store output in COLVAR file every 50 steps
PRINT ARG=* FILE=COLVAR STRIDE=50
```

Note that this input file is an example, and needs to be adjusted to be used, by replacing the labels a_0, b_0, a_n and b_m with the actual atom numbers of the atoms in the protein (labeled a) and the DNA (labeled b) included in the contact map. The input files as used in this work are provided on figshare: 10.6084/m9.figshare.c.6446950.

3 Sequence specificity of the ETS domain

3.1 Structure and function of ETS

The ETS protein family is one of the most ancient transcription factor families in animal evolution [3] and contains many different eukaryotic transcription factors with a highly conserved winged helix-loop-helix motif [11]. The ETS domain of the PU.1 transcription factor was the first structure to be resolved in complex with DNA, with X-ray crystallography, PDB-code 1PUE [7], revealing the ETS domain as a major groove binder. The ETS domain recognizes purine-rich sequences containing a 5'-(A/T)GGA(A/T)-3' consensus, and tolerates many variations among the flanking bases, as shown by equilibrium titration experiments [10]. The ETS protein PU.1 forms sequence-specific complexes with no fewer than 65 different sequences with a wide range of affinities [6, 9]. We selected the ETS domain from PU.1 to further test our simulation protocol, aiming to predict differences in binding of the ETS domain to three different nucleotide sequences. The nucleotide sequence from the crystal structure is also the consensus sequence as identified with equilibrium titration experiments [10] and alchemical calculations [4]: 5'-AAAAGGGGAAGTGGG-3', with only differences in the terminal base pairs. As a low affinity sequence, we inverted the consensus sequence by changing A to G and T to C, and vice versa: 5'-*CCCCTTTTCCTGAAA*-3'. Finally, we included an anti-consensus sequence for which experiments [10] and alchemical calculations [4] showed that the ETS domain has a lower affinity: 5'-AAAA**AA**GGAAG**GT**GG-3'. Supplementary Figure S6 shows snapshots of the systems and the three nucleotide sequences investigated in this work.

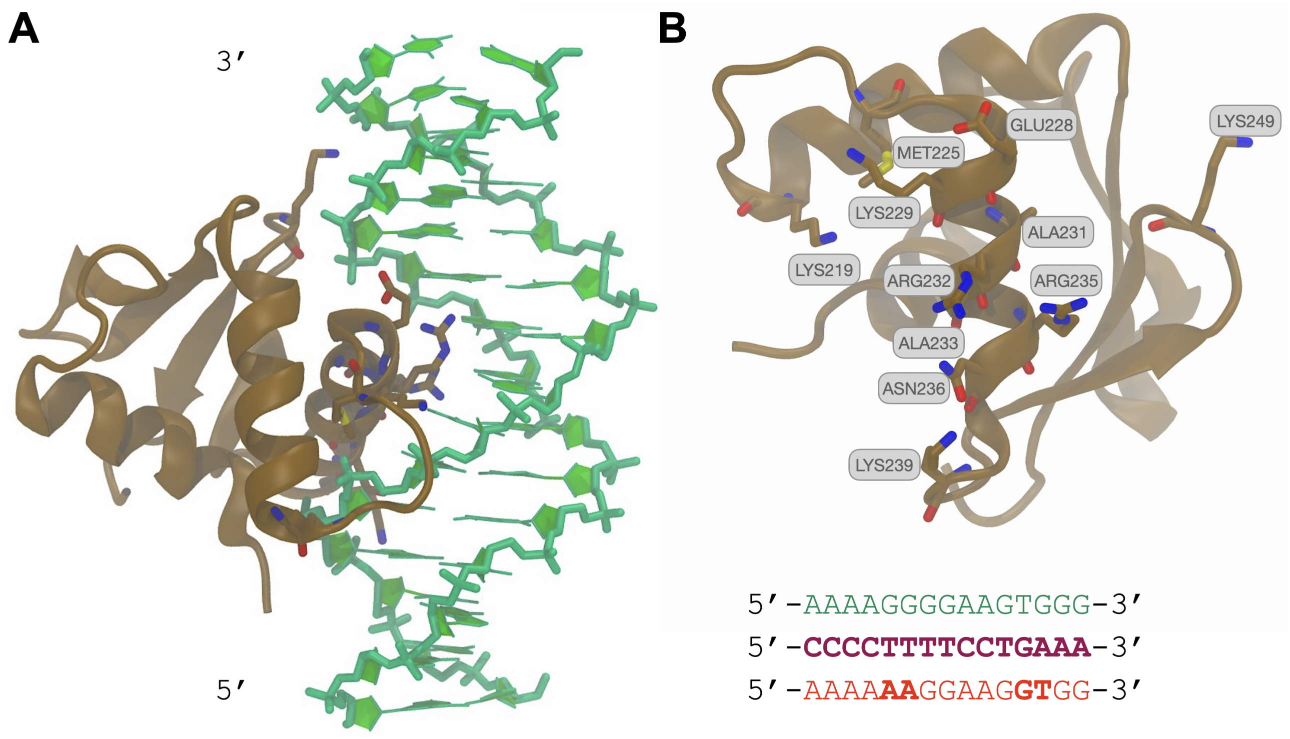

Supplementary Figure S6: A) Molecular representations of the ETS domain (brown) bound to the consensus sequence (green) with the DNA interacting amino acids shown as atomic licorice representations. B) ETS domain (brown) with labeled amino acids that are used for in the contact map for the analysis and steered molecular dynamics pulling coordinate as well as the three nucleotide sequences used in this work are shown in text: consenus (green), inverse (purple), and anti-consensus (red).

3.2 Characterization of the ETS-DNA complexes

As starting point for the simulation, we used the ETS domain from the PU.1 transcription factor in complex with DNA with the consensus sequence 5'-AAAAGGGGAAGTGGG-3', PDB code 1PUE [7], with the single stranded overhang residues removed. This sequence differs from the consensus sequence as identified in Ref. [10] in the terminal base pairs. For each protein-DNA complex we performed 4x 1 μs Molecular Dynamics simulations using the same protocol for the H-NS system as described in the methods section, with the exception that we used here a concentration of 150 mM NaCl to match *in vitro* experimental conditions [10].

Analysis of the MD simulations is similar as described in the main text, with the following adaptations to the contact map calculation. First, we identified the atom furthest from the alpha carbon (C_α) atom in the side chain of each amino acid residue in the protein. This atom served as a representative of the side chain for subsequent analyses. For all the DNA bases, we selected the N1 or N3 atoms based on whether the base was a pyrimidine or purine, respectively. These atoms were chosen as they are located at the core of the groove or near the helical axis of the DNA. We computed the distances between the representative atoms of the protein side chains and all DNA bases. Based on these distances we applied the switching function described in the methods section to obtain the number of contacts each protein residue has with the DNA. Then we summed the number of contacts each amino acid has with the DNA bases and normalized all values. Using the normalized contact count we proceeded to select only the amino acids that have higher count than 0.4, which resulted in the selection of 11 amino acids that form at the interface of the ETS and the dsDNA strand, shown in Supplementary Table S1. Next, we selected for each amino acid the hydrogen bond donor and acceptor atoms to construct a contact map between the N1/N3 atoms of the DNA sequence.

Supplementary Figure S7 shows the total contact count of the contact map for the cumulative data of the MD simulations for the three systems. See the previous paragraph for a description of the contact map calculation. The consensus and anti sequence show a bimodal distribution at 85 and 100 contacts, while the inverse sequence shows a uni-modal distribution around 85 contacts. In addition, we decomposed the total number of contacts by the individual contributions of the protein residues, which shows that ARG232 makes the most contacts with a count above 20 with the DNA. The anti-consensus sequence shows more variation for the ARG232 indicated by the second shoulder below 20 contacts. Overall, no significant difference between the sequences are observed, except that ARG235 makes more contacts with the DNA in the consensus sequence, compared to the inverse and anti sequences. In addition, the LYS249 has a bimodal distribution in which the peak at around 3 contacts has a higher maximum contact count binding mode for the consensus and anti sequence, but not for the inverse sequence. We also compared where the protein residues bind to the DNA by computing the median contact count for each residue with respect to the individual bases pairs of the DNA, which are shown as heat maps in Supplementary Figure S7C. The majority of the residues are

Supplementary Table S1: Contact map atoms between ETS and DNA grooves.

ETS		DNA	
Residue	Atoms	Base	Atom
LYS219	N, NZ	DA	N1
MET225	N	DT	N3
GLU228	N, OE1, OE2	DC	N3
LYS229	N, NZ	DG	N1
ALA231	N		
ARG232	N, NE, NH1, NH2		
ALA233	N		
ARG235	N, NE, NH1, NH2		
ASN236	N, OD1, ND2		
LYS239	N, NZ		
LYS249	N, NZ		

located in the helix that is bound to the major groove of the DNA. The two flanking residues LYS219 and LYS249 interact with the backbone region of the minor grooves in the 5' and 3' direction. The heat maps in Supplementary Figure S7 show that the arginines in the helix-loop-helix motif form the most contacts with the center of the nucleotide sequence, and the flanking lysines interact with the base pairs close to the DNA ends.

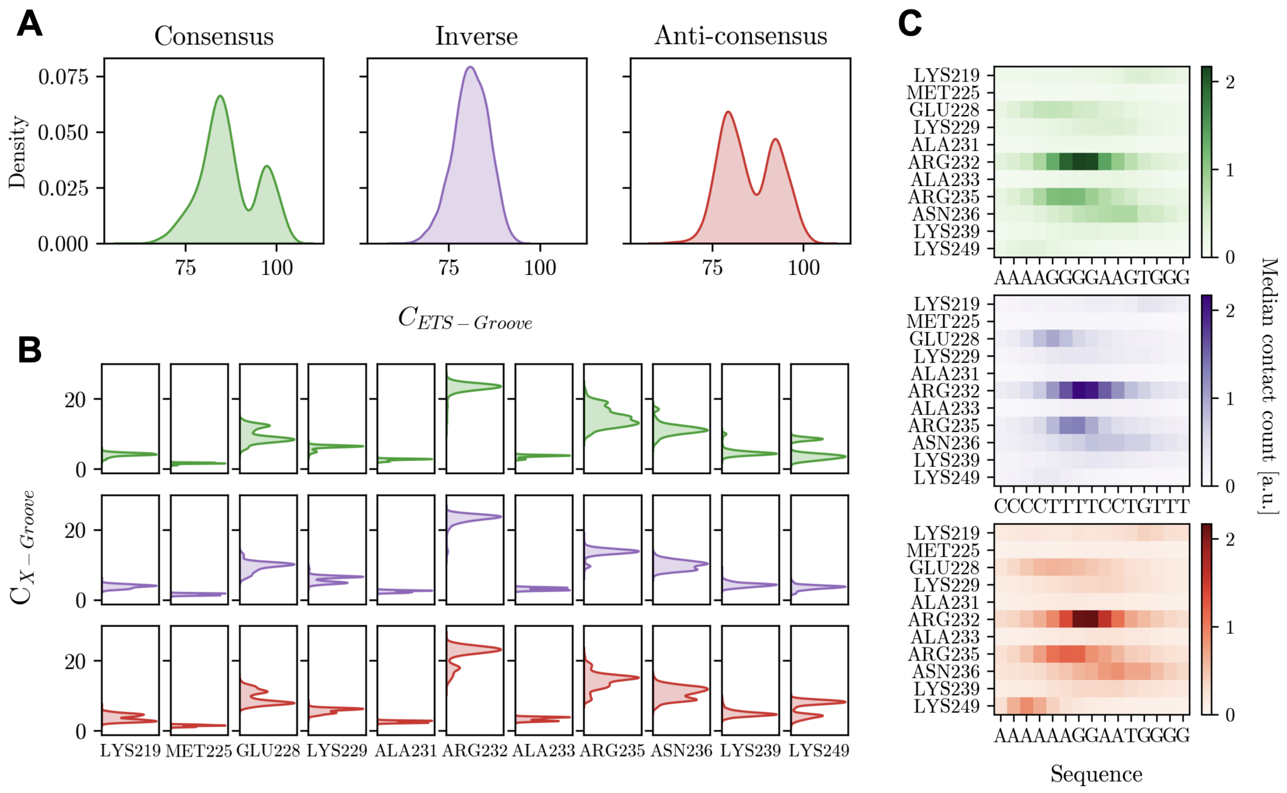

Supplementary Figure S7: A) Cummulative density estimates of the contact count between the ETS domain and DNA for the MD simulations of the consensus (green), inverse (purple) and anti-consensus (red) sequence. B)) Decomposed density estimates of the number of contacts of each ETS residue with respect to the complete DNA. C) Heat-maps of mean contacts of the ETS residues interacting with the basepairs of the respective sequences.

3.3 Nucleotide specificity of the ETS domain

As described in the methods section of the main text we transformed the contact map to a linear contact map (lcmap) to use as pulling coordinate during the steered molecular dynamics (SMD) simulations. Due to the higher number of residues included in the ETS systems only the 12 nearest nucleobases for each residue were included in the contact map. As the number of pairwise computations increases with a factor equal to the number of atoms when adding one extra atom to the contact map, this reduction in atom pairs made the SMD simulations much more efficient, enabling about 300 ns per day on a modern mixed GPU/CPU system (i.e. RTX 3090 with 32 AMD CPUs). Also, the cut-off range contributions of long-range interactions , i.e., bases further away, to the total contact count are negligible. We made the selection of atom pairs based on the consensus sequence and mapped these atom pairs to the other two sequences to have uniform comparison between the systems.

For the starting configurations we selected frames with a lcmap count of 210, which coincides with the highest populated modes at 85 contacts in Figure S7. We pulled from 210 (λ=1) to 120 (λ=0) contacts. We used the same SMD settings as for the H-NS/DNA systems: for the simulation time per run of 100 ns and a force constant of 500 kJ/mol nm^2 and performed 30 SMD runs. The PMF was computed as the average and the exponential average of the work of each run (see Methods section in the main text), and shown in Supplementary Figure S8. To illustrate the dissociation process, Supplementary Figure S8-B shows snapshots of the lowest work run of the consensus sequence at λ = 1.0, 0.5 and 0.0. At the end point of the SMD simulations, no sequence specific interactions with the major groove are present anymore. Comparing the ΔW values results in the following trend from highest to lowest binding affinity: consensus > anti-consensus > inverse. The $\Delta\Delta W = \Delta W_{Con} - \Delta W_{Anti/Inv}$ between the consensus and anti-consensus sequences is 2.57 kcal/mol and the difference between the consensus and inverse sequences is 3.87 kcal/mol. These values are within the expected difference shown in experiments, which showed that changing flanking bases lowers the binding affinity with 2.83 kcal/mol, in agreement with titration experiments [10] and alchemical calculations [4], see Supplementary Table S2. Directly comparing the ΔW from our work to the ΔG from the experiments shows that the values are in the same order of magnitude. Differences can be attributed to the choice of force field, which overestimate the binding energies, in particular of highly charged systems [14]. However, this would result in a systematic error that and not an increase in statistical errors. Our results also show that inverting the consensus sequence leads to even weaker binding than the anti sequence.

Supplementary Table S2: Reference values between our work, experimental measurements [10] and alchemical calculations [5] in kcal/mol.

Name	Sequence	ΔW	$\Delta\Delta W$	ΔG_{exp}	$\Delta\Delta G_{exp}$
Con.	`5'-AAAAGGGGAAGTGGG-3'`	20.63	-	11.69	-
Anti.	`5'-AAAAAAGGAAGGTGG-3'`	18.06	+2.57	8.86	+2.83
Inv.	`5'-CCCCTTTTCCTGAAA-3'`	16.73	+3.90	-	-

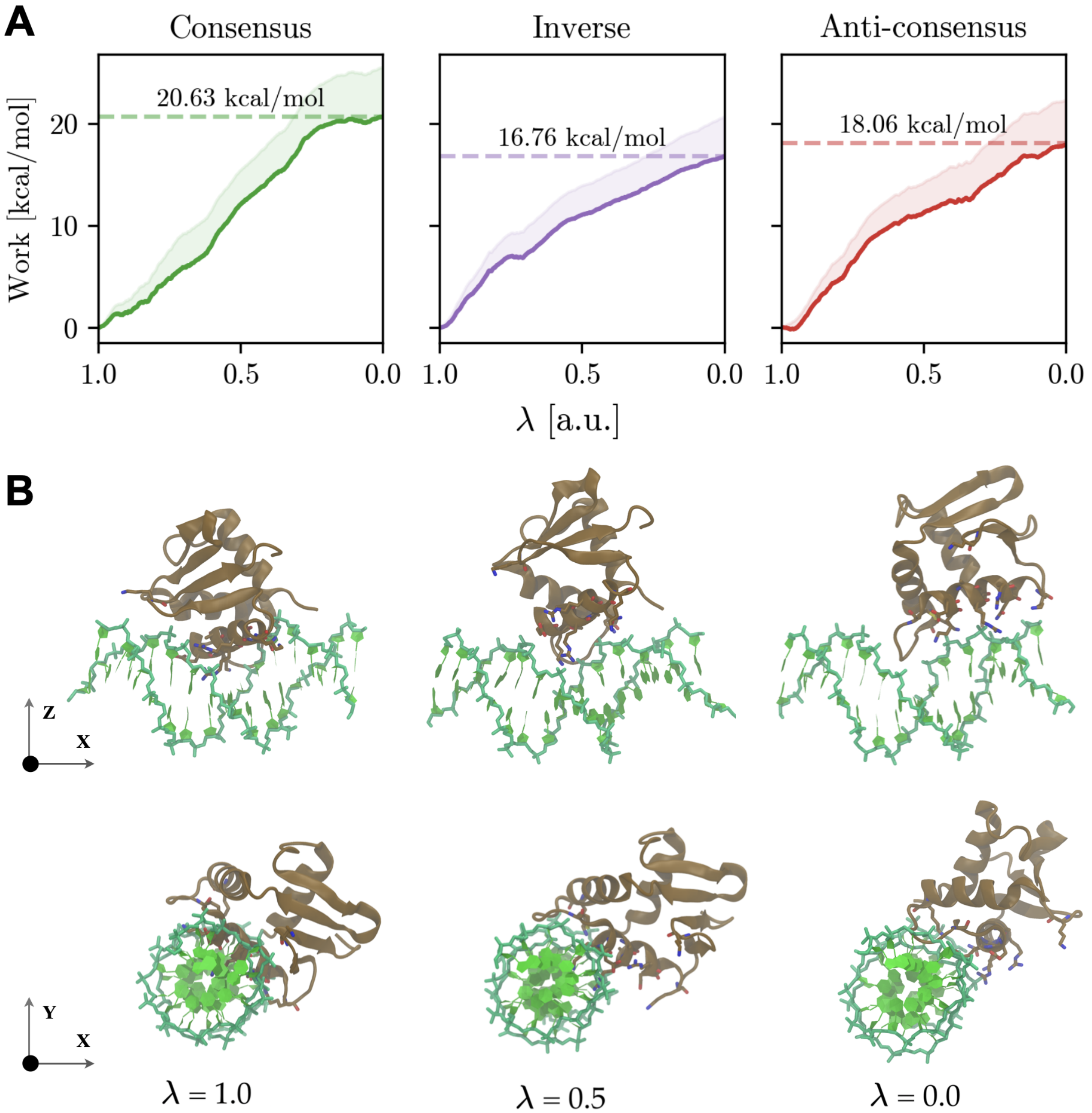

Supplementary Figure S8: A) PMF along $C_{ETS-groove}$ showing Boltzmann-weighted average work plot (solid lines) for each system (green consensus, purple inverse, and red anti-consensus). In addition with the standard deviation of all work-curves is shown with the shaded area above the Boltzmann-weighted average work. B) Shows snapshots along the progression of the pulling coordinate at λ = 1.0, 0.5, and 0.0 for the lowest work SMD run of the consensus affinity sequence starting in the FI state (with the dsDNA in green and the ETS domain in brown)

References

[1] Mark James Abraham et al. "GROMACS: High performance molecular simulations through multi-level parallelism from laptops to supercomputers". In: *SoftwareX* 1 (2015), pp. 19–25.

[2] The PLUMED Consortium. "Promoting transparency and reproducibility in enhanced molecular simulations". In: *Nature methods* 16.8 (2019), pp. 670–673.

[3] B.M. Degnan et al. "The ets multigene family is conserved throughout the Metazoa". In: *Nucleic Acids Research* 21 (1993), pp. 3479–3484.

[4] V. Gapsys and B. de Groot. "Alchemical free energy calculations for nucleotide mutations in protein–DNA complexes". In: *J. Chem. Theory Computation* 13 (2017), pp. 6275–6289.

[5] Vytautas Gapsys and Bert L de Groot. "Alchemical free energy calculations for nucleotide mutations in protein–DNA complexes". In: *Journal of Chemical Theory and Computation* 13.12 (2017), pp. 6275–6289.

[6] P. Gross et al. "Quantitative hydroxyl radical footprinting reveals cooperative interactions between DNA-binding subdomains of PU.1 and IRF4". In: *Biochemistry* 38 (1998), pp. 9802–9811.

[7] Ramadurgam Kodandapani et al. "A new pattern for helix–turn–helix recognition revealed by the PU. l ETS–domain–DNA complex". In: *Nature* 380.6573 (1996), pp. 456–460.

[8] Richard Lavery et al. "Conformational analysis of nucleic acids revisited: Curves+". In: *Nucleic acids research* 37.17 (2009), pp. 5917–5929.

[9] S. L. Li et al. "Critical flanking sequences of PU.1 binding sites in myeloid-specific promoters". In: *J. Biol. Chem.* 274 (1999), pp. 32453–32460.

[10] G.M.K. Poon and R.B. MacGregor Jr. "Base Coupling in Sequence-specific Site Recognition by the TeS domain of Murine PU.1". In: *J. Mol. Biol.* 328 (2003), 805=819.

[11] A.D. Sharrocks. "The ETS domain transcritpion factor family". In: *Nature Reviews* 2 (2001), pp. 827–837.

[12] Gareth A Tribello et al. "PLUMED 2: New feathers for an old bird". In: *Computer physics communications* 185.2 (2014), pp. 604–613.

[13] David Van Der Spoel et al. "GROMACS: fast, flexible, and free". In: *Journal of computational chemistry* 26.16 (2005), pp. 1701–1718.

[14] S. You et al. "Improved Parameterization of Protein–DNA Interactions for Molecular Dynamics Simulations of PCNA Diffusion on DNA". In: *J. Chem. Theory Comput.* 16 (2020), p. 4006.
